# Fish Skin Microbiomes Are Highly Variable Among Individuals and Populations but Not Within Individuals

**DOI:** 10.3389/fmicb.2021.767770

**Published:** 2022-01-21

**Authors:** Hanna Berggren, Petter Tibblin, Yeşerin Yıldırım, Elias Broman, Per Larsson, Daniel Lundin, Anders Forsman

**Affiliations:** ^1^Ecology and Evolution in Microbial Model Systems (EEMiS), Department of Biology and Environmental Science, Linnaeus University, Kalmar, Sweden; ^2^Department of Ecology, Environment and Plant Sciences, Stockholm University, Stockholm, Sweden; ^3^Baltic Sea Centre, Stockholm University, Stockholm, Sweden

**Keywords:** community ecology, diversity, fish, heterogeneity, repeatability, richness, skin microbiota, spatial variation

## Abstract

Fish skin-associated microbial communities are highly variable among populations and species and can impact host fitness. Still, the sources of variation in microbiome composition, and particularly how they vary among and within host individuals, have rarely been investigated. To tackle this issue, we explored patterns of variation in fish skin microbiomes across different spatial scales. We conducted replicate sampling of dorsal and ventral body sites of perch (*Perca fluviatilis*) from two populations and characterized the variation of fish skin-associated microbial communities with 16S rRNA gene metabarcoding. Results showed a high similarity of microbiome samples taken from the left and right side of the same fish individuals, suggesting that fish skin microbiomes can be reliably assessed and characterized even using a single sample from a specific body site. The microbiome composition of fish skin differed markedly from the bacterioplankton communities in the surrounding water and was highly variable among individuals. No ASV was present in all samples, and the most prevalent phyla, Actinobacteria, Bacteroidetes, and Proteobacteria, varied in relative abundance among fish hosts. Microbiome composition was both individual- and population specific, with most of the variation explained by individual host. At the individual level, we found no diversification in microbiome composition between dorsal and ventral body sites, but the degree of intra-individual heterogeneity varied among individuals. To identify how genetic and phenotypic characteristics of fish hosts impact the rate and nature of intra-individual temporal dynamics of the skin microbiome, and thereby contribute to the host-specific patterns documented here, remains an important task for future research.

## Introduction

Variation in fish skin microbiome has mainly been attributed to host species, geographic distribution and diet (Smith et al., 2007; Wang et al., 2010; Larsen et al., 2013, 2015; Stevens and Olson, 2015; Webster et al., 2018; Sylvain et al., 2020), but there also exists substantial variation in microbiome composition among individuals within species that remains elusive (Ross et al., 2019). A Web of Science topic search conducted on January 5th, 2021, showed that the scientific output on fish skin-associated microbiomes is growing rapidly, with a six-fold increase in annual output during the past two decades, from <100 contributions before 2000 to nearly 600 contributions published in 2020 (Supplementary Figure 1). Although the skin microbiome is of putatively significant ecological value to their host by aiding pathogen resistance (McLaren and Callahan, 2020) few studies have been conducted at the level of host individuals (Harrison et al., 2019; Ross et al., 2019) (but see recent contributions by Uren Webster et al. (2020) and Berggren et al., under review).

Earlier work on different host-microbe systems indicates that microbiome community compositions vary among host individuals as a consequence of ecological filtering imposed by a combination of host characteristics and environmental factors (Ley et al., 2008; Costello et al., 2012; Boutin et al., 2014; Sylvain et al., 2016; Miller et al., 2018; Krotman et al., 2020), and that this filtering is highly dynamic (Youngblut et al., 2019; Uren Webster et al., 2020). For instance, assembly processes within host species may vary according to life stage (Burns et al., 2016; Chiarello et al., 2019; Risely, 2020), host diet (Muegge et al., 2011), and genetics (Boutin et al., 2014; Webster et al., 2018). As such, assembly processes might vary according to the scale studied (Leibold et al., 2004). If fish are considered habitat islands that vary according to properties of the individual host and the environment it is experiencing, it might be hypothesized that the assembly processes operating within hosts would be more similar than among hosts. However, previous studies of skin microbiome have pointed to high variation in microbiome composition, both among individuals and according to bodily regions (Ley et al., 2008; Costello et al., 2009; Chiarello et al., 2015; Lowrey et al., 2015). The latter can potentially arise due to intrinsic factors such as host secretion and auto-immune molecules like defensins (Boutin et al., 2013; Chen et al., 2018), but extrinsic factors such as differential exposure to temperature, pH, sediments or light levels may also be involved (Shephard, 1994; Wotton, 2004; Grice and Segre, 2011; Wahl et al., 2012; Beck and Peatman, 2015; Hess et al., 2015). Ultimately this could also contribute to high inter-individual variability (Costello et al., 2009), given that patchy environments are expected to harbor higher species richness than homogeneous environments (Johnson and Simberloff, 1974; Tews et al., 2004). Moreover, differences in niche utilization due to behavioral variation expose host individuals to different microhabitats – even within the same population (Van Valen, 1965; Svanbäck and Bolnick, 2007; Forsman, 2015; Nordahl et al., 2018), and this may result in different microbiome composition among host individuals. At the level of host populations, it can be hypothesized that microbiome differences might be even larger due to localities varying in environmental conditions (Östman et al., 2010).

Although the processes shaping fish skin microbiomes attract increasing scientific attention (Supplementary Figure 1), few attempts have been made to evaluate how variation within and among fish individuals contribute to microbiome diversity within fish species. To evaluate the partitioning and respective contribution of alpha- and beta-diversity to the overall gamma-diversity requires multiple sampling scales, but this is seldom included in studies of microbial communities (Leibold et al., 2004; Sarkar et al., 2020; Walters and Martiny, 2020).

To investigate the partitioning of microbiome diversity within (alpha diversity) and among (beta diversity) host individuals and populations (gamma diversity), we sampled dorsal and ventral body parts from 39 individuals of perch (*Perca fluviatilis*) originating from two distinct populations along the Baltic Sea Swedish coast. Perch is a predatory fish species widely distributed in fresh- and brackish waters in the northern hemisphere of substantial socioeconomic value as a popular target in recreational fisheries (Craig, 2000; Tibblin et al., 2012; Donadi et al., 2017). We aimed to answer the following questions: (i) Do different fish host individuals, and different body parts within hosts, harbor microbiome communities that differ in species richness or community composition? (ii) Do microbiome community diversity and composition differ between different host populations? (iii) Does measurement repeatability allow the fish skin microbiome community composition to be reliably characterized using a single sample from each host individual?

## Materials and Methods

### Study System

Perch was sampled at two locations (Kalmar, 56°40.306′N; 16°21.578′E and Figeholm, 57°22.321′N; 16°33.340′E) separated by a swimming distance of approximately 80 km along the southeast Baltic coast of Sweden. This spatial separation exceeds the general dispersal pattern and home ranges (∼20 km) of Baltic Sea perch (Craig, 2000; Ahlbeck Bergendahl et al., 2017; Hansson et al., 2019) such that the locations likely harbor specific host populations with non-overlapping home ranges. This is also supported by comparisons of microsatellite data from Baltic Sea perch, including our study area, that show genetic clusters (populations) at a much finer spatial resolution than our two locations (Bergek and Björklund, 2009; Olsson et al., 2011). The spatial separation of populations also implies exposure to different environmental conditions that may shape the microbial community, such as sediment load, bottom substrates, and habitat heterogeneity (Caporaso et al., 2011; Walters and Martiny, 2020).

The Figeholm population resides in a habitat that consists of an approximately 5 km wide archipelago with distinct depth gradients as well as two streams discharging into the area. The drainage area is sparsely populated (<1,000 inhabitants), semirural and covered by coniferous forest suggesting limited direct anthropogenic impacts on the aquatic habitat. The Kalmar perch population inhabit a coastal area that generally lacks adjacent archipelago and freshwater inflows. The sample location is in the heart of the urban area of Kalmar municipality (>50,000 inhabitants) with substantial man-made modifications of the habitat including embankment, beachfront buildings and extensive hard surfaces resulting in direct anthropogenic impacts on the habitat and water quality.

### Sampling Procedures

Focal individuals [Kalmar: *n* = 30; Figeholm: *n* = 9; average size of 33.3 ± 3.6 cm (mean ± SD)] were captured between the 9th and 16th of October 2013. To avoid cross-contamination among individual fish microbiomes, individuals were captured one-by-one using rod and reel fishing. Microbiome samples were obtained from fish immediately after capture following rinsing with sterile MQ-water to eliminate bacterial cells associated with the water column. Pre-defined spots (approximately 2 × 2 cm) on the fish body were sampled with a sterile cotton swab that was twirled four times on the spot and then transferred to an Eppendorf-tube with 750 μl TE buffer (Tris-EDTA, 10:1). To minimize cross-contamination among samples all equipment used for sampling was sterilized with 70% ethanol between each sample. All samples were kept on ice until being stored at −80°C. The majority (32 of 39) of the fishes were females. Immediately after sampling, each fish was stunned and terminated by cervical dislocation. All applicable national guidelines for the care and use of animals were followed. Ethical approval for the study was granted by the Ethical Committee on Animal Research in Linköping, Sweden (Dnr. 33–14 and 10–14).

**FIGURE 1:**
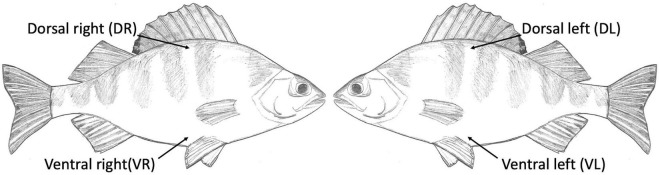
Body sites of *Perca fluviatilis* that were sampled to characterize variation in skin-associated microbiomes within and among host individuals. Dorsal (D) and ventral (V) samples from right (R) and left (L) side of the body. DR and VR sites were sampled on all 39 individuals included in the study and a subset of these individuals (*n* = 16) were also sampled on DL and VL sites.

We obtained two samples (dorsal and ventral) from the right side of every individual (*n* = 39). From a subset (*n* = 16) of these individuals, representing both populations, we also took two additional corresponding samples from the left side of the body (both dorsal and ventral) to be able to evaluate heterogeneity and sample repeatability within individuals (Figure 1). Data thus consists of 110 samples that represent 2–4 samples from 39 fish individuals originating from 2 different populations. Specifically, 82 samples from the Kalmar population of 30 fish individuals that were caught on three occasions (9, 11, and 15 October 2013; 4 samples from 11 individuals and 2 samples from 19 individuals) and 28 samples from the Figeholm population distributed among 9 fish individuals that were caught on one occasion (16 October 2013; 4 samples from 5 individuals and 2 samples from 4 individuals).

At each sampling occasion and location, surface water samples (1 L) were taken to enable comparisons of the microbial communities in the water column with those present on the fish skin. Water samples (*n* = 4) were vacuum filtered through a Supor membrane filter (Pall Corporation, pore size 0.22 μm, Ø 47 mm). The filter was transferred to a sterile tube containing 1.8 mL TE-buffer (Tris-EDTA, 10:1) and stored at −80°C until DNA extraction.

### DNA Extraction, Library Preparation, and Amplicon Sequencing

DNA was extracted from the microbiome samples collected with sterile swabs (*n* = 110) using the QIAmp DNA Mini Kit (QIAGEN, Germany) starting from step 3 in the protocol by the manufacturer. Final elution volume was set to 100 μl to increase the DNA yield. To minimize cross-contamination of samples, the nipper used to take out swabs was sterilized with 70% ethanol between each sample. DNA from water filters (*n* = 4) was extracted with the DNeasy PowerWater kit (Qiagen, Germany) and obtained concentrations were measured using NanoDrop 2000. Sequencing libraries were prepared by using the 16S rRNA gene primer pair 341F and 805R (Herlemann et al., 2011) following the PCR-protocol by Hugerth et al. (2014) modified by Lindh et al. (2015), with the exception of an additional five cycles in the first PCR. The process of adding Illumina adapters and index sequences was conducted according to Lindh et al. (2015). Final DNA concentrations were measured with a Qubit^®^ 2.0 Fluorometer. The resulting purified (individually barcoded) amplicons were pooled into three libraries that were purified with gel purification kit (E.Z.N.A.^®^ Gel purification kit, Omega Bio-Tek, Inc.) and sequenced on three separate occasions (*n* = 70 and 40 on each occasion for microbiome samples, and *n* = 4 for water samples) on the Illumina MiSeq platform (Illumina, United States) with 2 × 300 bp paired-end settings at Science for Life Laboratory (SciLifeLab, Stockholm, Sweden).

### Sequence Data Processing

#### Microbiome Samples

Raw sequences from each library were processed separately using the DADA2 package implemented in QIIME2 2018.8 with default settings except for the parent over abundance parameter that was set to four (Caporaso et al., 2010; Callahan et al., 2016, 2017; Bolyen et al., 2019). Merging of pair end reads was not possible due to high frequency of reads with low quality ends. Therefore, only forward reads were retained for analysis, an approach that has been used previously (Checinska et al., 2015; Videvall et al., 2018). Truncation lengths were set to 120 nucleotides (nt) and primers were trimmed by cutting 21 nt in the start of the sequence. By default, DADA2 corrects Illumina sequencing errors, remove chimeric sequences, and finally produces sequences with single-nucleotide resolution called “amplicon sequence variants” (henceforth ASVs). Sequences from both libraries were then combined into a single fasta file in RStudio (v1.3.1093) (R Core Team, 2013; RStudio Team, 2019). Taxonomy was assigned using a naïve Bayesian classifier trained on the V3–V4 region of the 16S rRNA gene with reference sequences (also truncated at 120 nt) from the SILVA database [SILVA v132; (Quast et al., 2013) in QIIME2 (v2019.10)]. Taxonomically assigned mitochondric sequences were filtered as possible contaminant DNA from the fish. Sequences that were unassigned at the domain level were also filtered out. After pre-processing, 40,291,388 raw sequences were down to 30,308,550 sequences and 5,778 ASVs. The microbiome samples (*n* = 110) consisted of 275,532 sequences on average (median = 265,779; range = 25,787–1,007,839). According to rarefactions curves, the community members within samples are expected to be sufficiently covered (Supplementary Figure 2).

#### Water Samples

Raw sequence data from the sequencing occasion including the water samples was processed independently using the DADA2 package in QIIME2 2018.8. All settings were the same as for the microbiome samples, except that merging of pair-end reads was successful. This means that ASVs from microbiome and water samples had different lengths and thus comparison at the level of ASVs was impossible. However, taxonomic comparison was still possible. After pre-processing the water data (*n* = 4) consisted of 55,362 sequences and 1,278 ASVs.

### Statistical Analyses

#### Richness Measurements (Alpha Diversity)

All statistical analyses were performed in Rstudio (v1.3.1093) (R Core Team, 2013; RStudio Team, 2019) unless stated otherwise and the code is provided in Supplementary Data File 1. To quantify richness within individuals we used both the number of observed ASVs, and estimated the species richness using the “breakaway” package (v4.6.11) with default settings (Willis, 2019). This function estimates richness from a non-linear regression model based on probability theory and the observed frequency counts. To evaluate whether the number of samples from each individual affected the observed richness, we performed a one-way ANOVA on the groups that were sampled two and four times, respectively. The test was performed with *aov* function in the stats package under the null hypothesis that individuals represented by four samples would not display higher observed richness.

#### Community Composition Measurements (Beta Diversity)

Because of the compositional nature of data sets obtained from high throughput sequencing (HTS) (Gloor et al., 2017), we performed a centered log ratio (clr) transformation of the microbiome data (Aitchison et al., 2000) to make it symmetric and linearly related (Pawlowsky-Glahn et al., 2015). First, we calculated point value estimates with zCompositions package (v1.2.31) representing estimates of the probability for each observation in the data set to deal with the general zero-inflatedness in microbiome data (Gloor et al., 2017). Then, we performed clr-transformation on those probabilities with CoDaSeq package (v0.99.3). This method of estimating the probability of the observation to be a true observation does not only offer a solution to the compositional structure of the data, it is also a way to circumvent the differences in number of sequences among samples that is usually solved by rarefying (McMurdie and Holmes, 2014). The clr-transformed values can be used as input for both multivariate analysis, such as (PER)MANOVA, and regressions (e.g., constrained redundancy analysis) since the log-ratios makes the data symmetric and linearly related (Pawlowsky-Glahn et al., 2015).

#### Exploring the Effects of Host Population and Individuals on Microbiome Composition

To test for effects of host population and individuals on the composition of the microbiome we performed constrained redundancy analyses (henceforth RDA) with the *rda* function implemented in the vegan package (v2.5-6) (Dixon, 2003; Oksanen et al., 2019) which uses ordinary unweighted linear regression on constraining variables. To evaluate model fit we used the function *anova.cca*, which is a permutation-based test that allows for nesting of factors. Since microbiome sequences were produced on two separate sequencing runs (i.e., different MiSeq flow cells), we restricted permutations within each flow-cell when evaluating effects of population and individuals (*n* = 110). Adjusted R-squared values were calculated with function *RsquareAdj* in the vegan package.

#### Evaluating Heterogeneity and Intra- vs. Inter-Individual Variation in Microbiome Composition

To determine the repeatability of samples taken from mirroring left and right side of dorsal and ventral body sites, we used samples from 16 host individuals that contributed with four samples (*n* = 64) and performed an intraclass correlation analysis on the estimated species richness. This was done with the *ICC* function in the R package psych (v1.8.12) (Revelle, 2019), using the ICC1 option as outlined by Shrout and Fleiss (1979) that includes a one-way ANOVA fixed effects model based on (MSB–MSW)/[MSB + (nr − 1) × MSW].

To explore the partitioning of the variance among samples from different levels, we used the function *betadisper* in the vegan package with Euclidean distances as input. We extracted the distance to the group centroid for each of the following levels: each body site within individuals (dorsal and ventral separately); within individuals (both dorsal and ventral); among individuals; and between populations.

**FIGURE 2:**
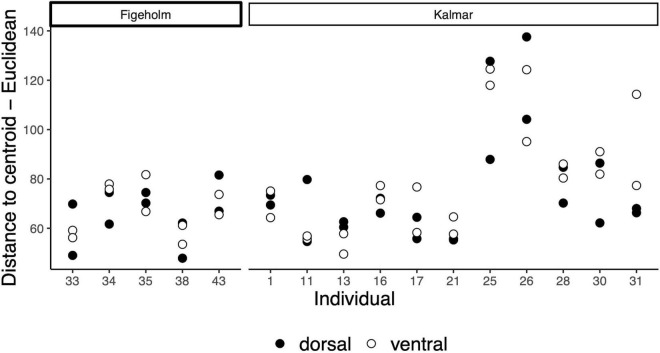
Variance among replicate samples of fish skin microbiomes from dorsal and ventral body sites within different host individuals. Beta dispersion plots based on Euclidean distances to centroid. Each dot represents one sample and shows the dispersion among the four samples taken from mirroring left and right sides of the dorsal and ventral body sites from each fish individual (*n* = 16) collected from Figeholm and Kalmar.

To assess whether the composition of the skin microbiomes was heterogeneous within individuals, we performed a PERMANOVA on Euclidean distance matrix in PRIMER-E v7 (Anderson et al., 2008) with 10,000 permutations. The test included 16 individuals that contributed with four samples each (*n* = 64). In this model, individual was set as a random factor to control for repeated sampling, and body site (dorsal or ventral) was treated as a fixed factor. We further performed PERMDISP as a complementary test to aid the interpretation of the results from the main tests. PERMDISP gives a *p*-value regarding the null hypothesis that dispersions around the mean or centroid are homogenous across samples (Anderson, 2006). Results revealed significant differences among hosts regarding heterogeneity of community composition, such that some hosts varied more in community composition between dorsal and ventral body parts than others (note individual 25 and 26 in Figure 2). Results from the main tests were qualitatively similar with or without outliers (PERMANOVA, Euclidean matrix, effect of host individual: *F*_13, 41_ = 2.47, *P* < 0.001, *R*^2^ = 0.39; effect of body site within host: *F*_1, 41_ = 0.92, *P* = 0.30, *R*^2^ = −0.03; PERMDISP, *F*_13, 42_ = 3.47, *P* = 0.11), thus we report on the results where outliers are included.

#### Data Exploration

The hierarchical diversity figure was generated with SigmaPlot for Windows (v12.5, Build 12.5.0.38). The rarefaction plot was generated in ampvis2 (Andersen et al., 2018) using function *amp_rarecurve*. All other plots were made with ggplot2 (Wickham, 2016) in Rstudio (R Core Team, 2013; RStudio Team, 2019).

## Results

**FIGURE 3:**
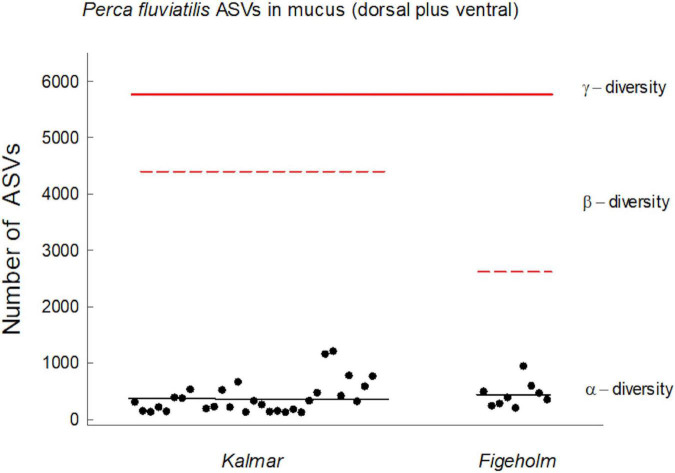
Hierarchical levels of fish skin microbiome richness representing host individuals, populations, and regional scales. Each dot represents an individual, black line represents the mean richness among individuals within Kalmar and Figeholm population, respectively. Red dashed line represents the total numbers of 16S rRNA gene ASVs observed within each population. Red solid line represents the total number of ASVs found among all samples (5,778 ASVs).

Total observed richness summed across all microbiome samples was 5,778 ASVs. The total richness found within each population was greater than mean richness observed among individuals, demonstrating that the microbiome community compositions varied among hosts (Figure 3). The varying number of ASVs found on each individual fish host (Figure 3) did not reflect differences in the number of samples collected from each individual such that there was no difference in the number of ASVs between individuals that were sampled four (*n* = 16) or two (*n* = 22) times, respectively (ANOVA: *F*_1, 37_ = 1.77, *P* = 0.19).

**FIGURE 4:**
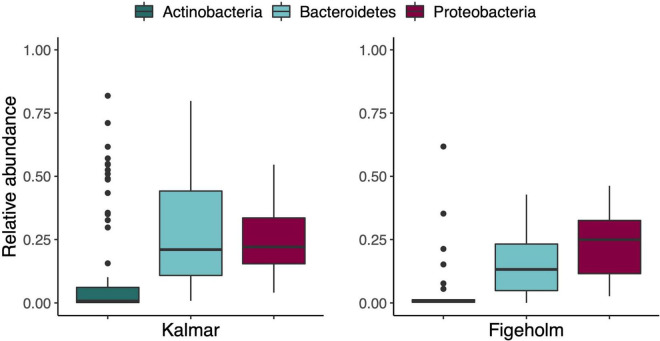
Relative abundance of the three most prevalent phyla among all microbiome samples, split by population. Boxplot elements: center line, median; box limits, upper and lower quartiles; whiskers, 1.5× interquartile range. Each dot represents a sample.

Only three of the 5,778 ASVs were detected in 80% of the samples and they belonged to families Burkholderiaceae (*Variovorax paradoxus*, and genera *Burkholderia*, *Caballeronia*, and *Paraburkholderia*) and Rhizobiaceae (genus *Ensifer*). The relative abundance of these two families varied between 0.04 and 29% and 0.002 and 1.1%. Only three phyla were present in all samples: Actinobacteria, Bacteroidetes, and Proteobacteria with ranges among samples varying between 0.003–82, 0.02–80, and 2.7–55%, respectively (Figure 4).

### Microbiome Composition Varied Among Host Individuals From Different Source Populations

**FIGURE 5:**
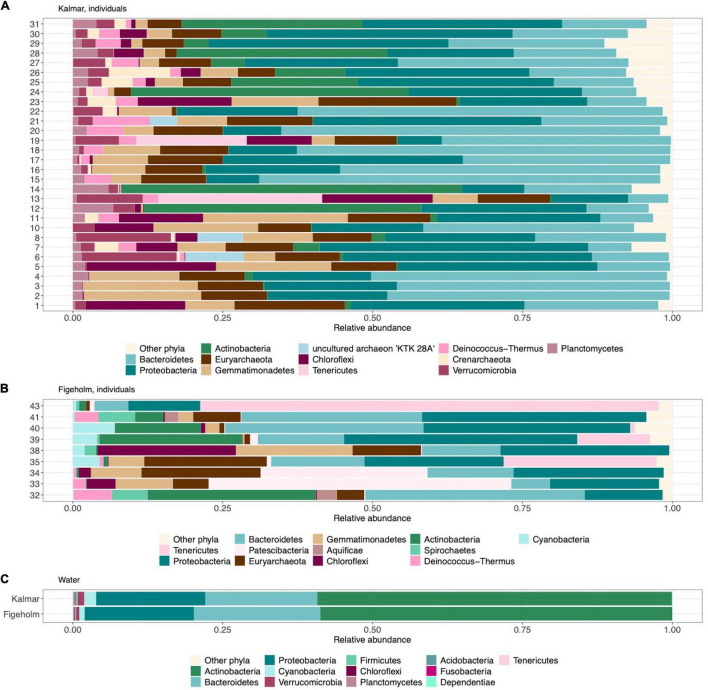
Comparison of phylum-level composition in fish skin microbiome and bacterioplankton communities, ordered by increasing relative abundance from left to right. The 12 most abundant phyla based on relative abundance among fish skin microbiome samples within **(A)** population Kalmar (*n* = 30 individuals), and **(B)** population Figeholm (*n* = 9 individuals), respectively. Each number on the y-axis denotes an individual fish represented by either 2 or 4 samples. **(C)** Displays taxonomic comparison of bacterioplankton communities in the water samples from Kalmar (*n* = 3) and Figeholm (*n* = *1*) and represent the 12 most abundant phyla among all water samples.

Both source population and individual accounted for variation in the microbiome composition, but host individual explained a larger proportion of the total variation than did population (RDA, *n* = 110, effect of population: *F*_1, 108_ = 1.80, *P* = 0.007, *R*^2^ = 0.007; effect of individual: *F*_38, 71_ = 1.76, *P* = 0.001, *R*^2^ = 0.21; Figures 5A,B and Supplementary Figure 3). The amount of variation in microbial community composition was comparable between the two populations (ANOVA, effect of population on Euclidean distance to centroid: *F*_1, 108_ = 0.046, *P* = 0.83), indicating that the difference in microbiome composition was not due to a difference in variance among samples within populations. When analyzing host populations separately, individuals within populations were still significantly different from each other (RDA, effect of individual, Figeholm: *F*_8, 19_ = 2.10, *P* < 0.001, *R*^2^ = 0.25; Kalmar: *F*_29, 52_ = 1.65, *P* < 0.001, *R*^2^ = 0.19).

The total number of phyla discovered among all fish skin microbiomes was 57, of which 52 were present in the Kalmar and 47 in the Figeholm population, respectively. Comparisons of the 12 most abundant microbiome phyla in each host population showed that 8 of these 12 phyla were shared between the two populations, but their prevalence varied greatly among individuals (Figures 5A,B). Moreover, these eight phyla included three phyla that were not detected in the water samples: Euryarchaeota, Deinococcus-Thermus, and Gemmatimonadetes. A few phyla had notably high mean relative abundance in one host population while being rare in the other, and this was attributable to variation among individuals. For example, the phyla Tenericutes (majority of sequences belonging to family Mycoplasmataceae) had mean relative abundances 28.7% ranging between 0.007 and 88% in Figeholm, but 4.4%, varying between 0.001–35% in Kalmar, and Patescibacteria (majority of sequences belonging to family Gracilibacteria bacterium JGI 0000069-P22) had mean relative abundance 14.4%, with a range of 0.002–80% in Figeholm while the mean relative abundance was 0.8%, ranging between 0.001 and 9.2% in Kalmar (Figures 5A,B). Within population comparisons revealed that except for the phyla present in all samples from both populations (Figure 4), Patescibacteria was present in all samples from the Kalmar host population. No other phyla were present in all samples from either of the two populations, respectively.

We identified a total of 1,278 ASVs in the bacterioplankton communities from water samples, distributed among 16 and 12 phyla in Kalmar and Figeholm, respectively. None of these phyla were exclusively found in water. The most abundant taxonomic groups, Actinobacteria, Bacteroidetes, and Proteobacteria, were shared between water samples from the two geographic locations, [overall mean abundance 29.4% (range = 18.4–58.7%); 9.9% (range = 4.3–21.1%); 9.1% (range = 4.8–18.2%)] (Figure 5C). These phyla were also the most abundant in skin microbiomes but included more clades at lower taxonomic levels (23, 13, and 87 vs. 13, 8, and 31 orders in microbiome and water samples respectively Supplementary Data File 2). The community composition of bacterioplankton communities in the water was significantly different from that in the skin microbiomes (RDA, effect of sample type: *F*_1, 112_ = 17.4, *P* < 0.001, *R*^2^ = 0.13; Figure 5).

### Evaluating Heterogeneity in Microbiome Community Composition Within Individuals

**FIGURE 6:**
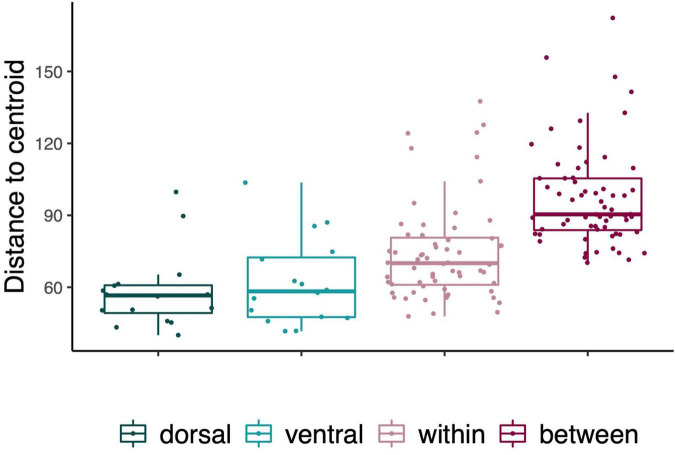
Intra- vs. inter-individual comparisons of variation among samples of fish skin microbiomes. Variation among samples is measured as Euclidean distances to centroid. Dorsal (petrol blue) and ventral (turquoise) boxes display pairwise comparisons of replicated samples within individuals (mirroring right and left side from each body site, respectively). The estimates of variation within individuals (pink) are based on the four samples from each of the 16 individuals, and the estimates of variation among individuals (plum) are based on all samples from the 16 individuals together. All data points are shown. Box-plot elements: center line, median; box limits, upper and lower quartiles; whiskers, 1.5× interquartile range.

Species richness was highly correlated between left and right samples for both dorsal and ventral body sites within individuals (dorsal: 85.6%, *F*_15, 16_ = 12.9, *P* < 0.001; ventral: 87.1%, *F*_15, 16_ = 14.5, *P* < 0.001, Figure 6). The microbiome community composition did not differ significantly between samples taken from the dorsal and ventral body parts of the same host individuals, but the significant variation among hosts as reported above (see the effects of individual in the RDA analyses in the previous section) was further supported (PERMANOVA random effect of host individual: *F*_15, 47_ = 2.16, *P* < 0.001, *R*^2^ = 0.22; effect of body site within host: *F*_1, 47_ = 1.11, *P* = 0.30, *R*^2^ = 0.03, Figure 6). There was a significant difference in variance among samples taken from the same host individual (PERMDISP: *F*_15, 48_ = 10.2, *P* < 0.001, Figure 2). This reflected that the microbiome community composition was more heterogeneous (i.e., differed more between body sites) in some host individuals than in others (note individual 25 and 26 in Figure 2).

## Discussion

In this study, we compared samples from different body sites, individuals and source populations of *P. fluviatilis*. The study offers the first attempt, to our awareness, to systematically evaluate intra-individual heterogeneity of fish skin microbiomes and investigate how variation in microbiome composition accumulates along hierarchical levels of organization. In brief, our results demonstrate that the community composition of the fish skin microbiomes was different overall from the microbial community in the surrounding water, did not differ between dorsal and ventral body sites within hosts, varied considerably among host individuals, and differed according to host population. Our analyses also uncovered a high similarity of microbiome samples taken from the left and right side of the same individuals, suggesting that fish skin microbiomes can be reliably assessed and characterized even using a single sample of host phenotypes.

### Taxonomic Patterns

We found more ASVs at the population than individual level (Figure 3), indicating that microbiome composition varied among individual fish hosts. When examining the prevalence of phyla, only three were present in all samples: Actinobacteria, Bacteroidetes, and Proteobacteria, and their relative abundances were highly variable among samples (Figure 4). These phyla were also present in the bacterioplankton communities in the water. However, their relative abundances were much higher in the water compared to the microbiome samples (Figure 5), and the composition at lower taxonomic levels was far more diverse in fish skin microbiomes (Supplementary Data File 1). Other studies have also found the three aforementioned taxa to be the most prevalent in fish skin microbiomes (Boutin et al., 2014; Chiarello et al., 2015; Legrand et al., 2018; Krotman et al., 2020; Uren Webster et al., 2020), indicating that these phyla include symbiotic and commensal bacterial taxa that thrive on fish skin (Tarnecki et al., 2019). Another interesting finding was that three of the most abundant phyla that were present in both populations (Euryarchaeota, Deinococcus-Thermus, and Gemmatimonadetes; Figures 5A,B) were unique to fish skin microbiome samples (i.e., not detected in water), which could point to a specific association to fish microbiome. These three phyla have previously been reported in studies associated with fish microbiomes. For instance Gemmatimonadetes has been reported in the gut of Atlantic salmon (*Salmo salar*) (Dehler et al., 2017), and Euryarchaeota was detected in the skin microbiome of coral reef fishes (Chiarello et al., 2018). Deinococcus-Thermus have been detected in certain parts of the gut of the yellow grouper (*Epinephelus awoara*) (Egerton et al., 2018), but also on the skin of the common snook (*Centropoumus undecimalis*) (Tarnecki et al., 2021), and it is noteworthy that both of these species belong to the same order as perch, Perciformes.

The three ASVs present in at least 80% of the fish skin samples belonged to the families Burkholderiaceae and Rhizobiaceae, both of which are commonly associated with fish skin (Reinhart et al., 2019; Chiarello et al., 2020; Uren Webster et al., 2020). One ASV was annotated to species level, *Variovorax paradoxus*, a bacterium that can utilize many different organic compounds (Willems et al., 1991), and thus possibly a commensal microbiome member of perch that feed on the nutrient rich mucus (Shephard, 1994).

Taken together, these findings are in accordance with previous studies on fish skin microbiomes that also report on high variability among individuals in microbiome composition (Chiarello et al., 2015; Legrand et al., 2018, 2020; Uren Webster et al., 2020), and that fish skin microbiome composition is different from the surrounding bacterioplankton (Horsley, 1977; Wang et al., 2010; Stevens and Olson, 2013; Chiarello et al., 2018, 2019; Krotman et al., 2020; Sylvain et al., 2020; Uren Webster et al., 2020). One possible explanation for the different composition in fish skin microbiome compared to water, is that the fish skin offers a nutrient rich habitat for epibiotic bacteria in terms of the mucosal layer (Ángeles Esteban, 2012). The mucus constitutes a diversity of gel glycoproteins that have been suggested to provide many ecological niches for microbial organisms (Shephard, 1994; Wotton, 2004; Brown and Bythell, 2005; Chiarello et al., 2015, 2018; Sylvain et al., 2016).

### Individual-Specific vs. Population-Specific Microbiome Composition

Although we found statistical support for microbiome differences between populations, host individual accounted for a larger proportion of the total variation in microbiome composition than did host population. Individual-specific variation was evident also when considering the most abundant taxonomic groups; a few phyla were over- and underrepresented in the respective populations (e.g., Tenericutes and Patescibacteria, Figures 5A,B), however, this skewed abundance was an effect of host individual rather than population. A potential explanation for the relatively low differentiation between the two host populations in our study is that the host species is a strong determinant of the associated microbial community composition (Larsen et al., 2013; Stevens and Olson, 2013; Chiarello et al., 2018; Sylvain et al., 2020), at least relative to the effects of the environment (Östman et al., 2010). This was suggested by Stevens and Olson (2015) with the argument that the fish host-microbiome interaction creates niches that potentially make the skin surface less accessible for free-living microorganisms and possibly more resistant to fluctuations in abiotic factors (e.g., pH, temp, and nutrients) than bacterioplankton communities (Pinhassi et al., 2003; Martiny et al., 2006). It has previously been reported that population differences in skin microbiome compositions are correlated with genetic dissimilarities (Webster et al., 2018), and this could potentially apply to the pattern observed here since perch populations in the different study sites are genetically distinct (Olsson et al., 2011).

However, the high individual variation in microbiome composition, reported in our study, might be indicative of the fact that stochastic processes influence which microbes that colonize the skin of perch (Burns et al., 2016; Chiarello et al., 2019). Alternatively, the large variation of microbiomes among individual hosts may reflect individual differences in the genetic make-up of the immune system (Boutin et al., 2014; Malmstrøm et al., 2016; Webster et al., 2018), diet preferences (Chiarello et al., 2018; Uren Webster et al., 2020), and behaviors (Bolnick et al., 2003). Host individuals can be regarded as islands with different properties according to both intrinsic (e.g., host genetic and phenotypic variation) and extrinsic factors (e.g., external environmental conditions in the habitat). This means that their skin-associated microbiomes are exposed to constantly changing, and possibly contrasting, environmental conditions–both from the hosts and the environment that the host is exposed to. These combined features result in environmental heterogeneity which might have the potential to promote more species rich and diverse communities at higher level of biological organization (microbial communities), as have been shown for trophic levels in other systems (e.g., Koricheva and Hayes, 2018; Raffard et al., 2019).

### The Degree of Intra-Individual Heterogeneity in Microbiome Composition Varied Among Individuals

In animal and plant ecosystems, heterogeneous or patchy environments are expected to promote species diversity (Johnson and Simberloff, 1974; Tews et al., 2004) and in this context, heterogeneity in microbiome composition within individuals can be of eco-evolutionary interest and importance (Anderson et al., 2006; Forsman and Wennersten, 2016; Yildirim et al., 2018). In line with this prediction, we hypothesized that different body parts constituted contrasting habitats and thus harbored different microbial communities (Costello et al., 2009; Chiarello et al., 2015). However, according to our results, the microbiomes on dorsal and ventral body sites within host individuals were not distinct from one another. This can either reflect similar microbiome assembly processes (Costello et al., 2009; Kraft et al., 2015), or that connectivity and dispersal of microbes between bodily regions within the host is high (MacArthur and Wilson, 1967; Miller et al., 2018).

Interestingly, we found that the within-individual variability of microbiome composition varied significantly among host individuals (see results from PERMDISP and Figure 2). This could reflect behavioral variation among host individuals. Vertical migration associated with foraging, thermoregulation and diel activity patterns expose the dorsal and ventral microbiomes to contrasting environmental conditions, and possibly also to different species pools of potential microbial colonizers (Bolnick et al., 2003; Nordahl et al., 2018, 2020). It can therefore be hypothesized that the difference between dorsal and ventral microbiomes should be more pronounced in individuals that engage in vertical migrations to a higher degree. Given that there is typically a large proportion of unexplained variation in microbiome composition across fish hosts (Falony et al., 2016; Miller et al., 2018), it is critical to evaluate the reliability and repeatability of microbiome samples taken from the same host individual. Knowledge about individual differences and measurement consistency and how they influence the partitioning of the total variance can inform sampling design, with potential to increase the reliability and to improve reproducibility of future studies (Voelkl et al., 2020). In our case, the high similarity of microbiomes sampled on the left and right side within perch individuals (Figure 6) suggests that the observed differences in variance between dorsal and ventral samples among individuals were not resulting from measurement error.

Previous attempts to sample, quantify and compare microbiomes between different functional parts of the fish host have not formally evaluated heterogeneity among body sites while accounting for individual identity using repeated samples from each body site (Chiarello et al., 2015; Lowrey et al., 2015; Legrand et al., 2018). The results from this study, based on repeated samples taken from the same individuals and body sites, thus provide novel insights on how variation among individuals in alpha diversity, and the degree of spatial heterogeneity within individuals, contribute to beta-diversity in fish skin microbiomes.

## Conclusion and Future Directions

Here we have reported on a study of microbiomes that inhabit the skin of perch showing that: (i) fish skin microbiomes are highly diverse, even at the level of phyla; (ii) fish individual accounts for a high proportion of the variation in microbiome composition; and that (iii) the microbiome composition is not patchy within individual fish, but the degree of heterogeneity varies among individuals. The results also indicated that fish skin-associated microbiomes can be sampled, quantified, and characterized with high repeatability at the studied body positions. There is currently a knowledge gap about what drives the spatiotemporal dynamics of microbiomes within individual fish hosts. Our present findings thus have implications for future studies in that they emphasize the need to consider individual-specific effects when attempting to disentangle the importance of extrinsic vs. intrinsic factors. Besides identifying the role of ecological filtering imposed by the environment, an important task for future research is to determine the genetic, phenotypic and behavioral characteristics of hosts that affect the assembly and dynamics of fish skin microbiomes, and that thereby contribute to the type of individual-specific patterns documented in this study. Other challenges for the future are to perform repeated longitudinal sampling of fish hosts to assess the rate and nature of intra-individual temporal dynamics of microbiomes and to investigate whether and how they change with host behaviors and habitat shifts.

## Data Availability Statement

The datasets generated in this study can be found in the NCBI SRA database under accession number PRJNA716301 and can be found here: https://www.ncbi.nlm.nih.gov/bioproject/PRJNA716301.

## Ethics Statement

The animal study was reviewed and approved by the Ethical Committee on Animal Research in Linköping, Sweden, Dnr. 33–14 and 10–14.

## Author Contributions

HB and AF conceived the study. HB, PT, PL, and AF designed the study. HB and PT conducted the field work. HB and YY performed the laboratory work. HB, EB, and DL performed the bioinformatics analyses. HB and YY performed the statistical analyses with support from AF. HB wrote the first draft. All authors contributed to interpreting the results, read and approved the final version of the manuscript.

## Conflict of Interest

The authors declare that the research was conducted in the absence of any commercial or financial relationships that could be construed as a potential conflict of interest.

## Publisher’s Note

All claims expressed in this article are solely those of the authors and do not necessarily represent those of their affiliated organizations, or those of the publisher, the editors and the reviewers. Any product that may be evaluated in this article, or claim that may be made by its manufacturer, is not guaranteed or endorsed by the publisher.

## Supplementary Material

Supplementary Figure 1 Publication trend on fish skin microbiome. A Web of Science topic search [using the search string (skin OR epiderm*) AND (microb* OR bacteri* OR microorg* OR microflora OR microbiome OR microbiota) AND fish*] conducted on 5th of January 2021 yielded 7,299 publications indicating that scientific output on fish skin-associated microbiomes is limited but growing by each year.

Supplementary Figure 2 Rarefaction curves for fish skin-associated microbiomes and bacterioplankton communities in water samples. Curves display a sufficient sequence coverage.

Supplementary Figure 3 PCA plot based on model output from rda analysis on population differences. All 110 samples are represented. Different colors represent individual (*n* = 39) and shape population affinity (triangles = Kalmar, circles = Figeholm). The plot is based on the output from the rda analysis with population as constraining variable.

Supplementary Data File 1 ASV-table and taxonomy.

Supplementary Data File 2 R-code for data processing, analysis and figures.
