# Supplementary figures and images for: Fish Skin Microbiomes Are Highly Variable Among Individuals and Populations but Not Within Individuals
Source: Front Microbiol. 2022 Jan 21;12:767770. doi: 10.3389/fmicb.2021.767770 (PMC8813977; doi:10.3389/fmicb.2021.767770)

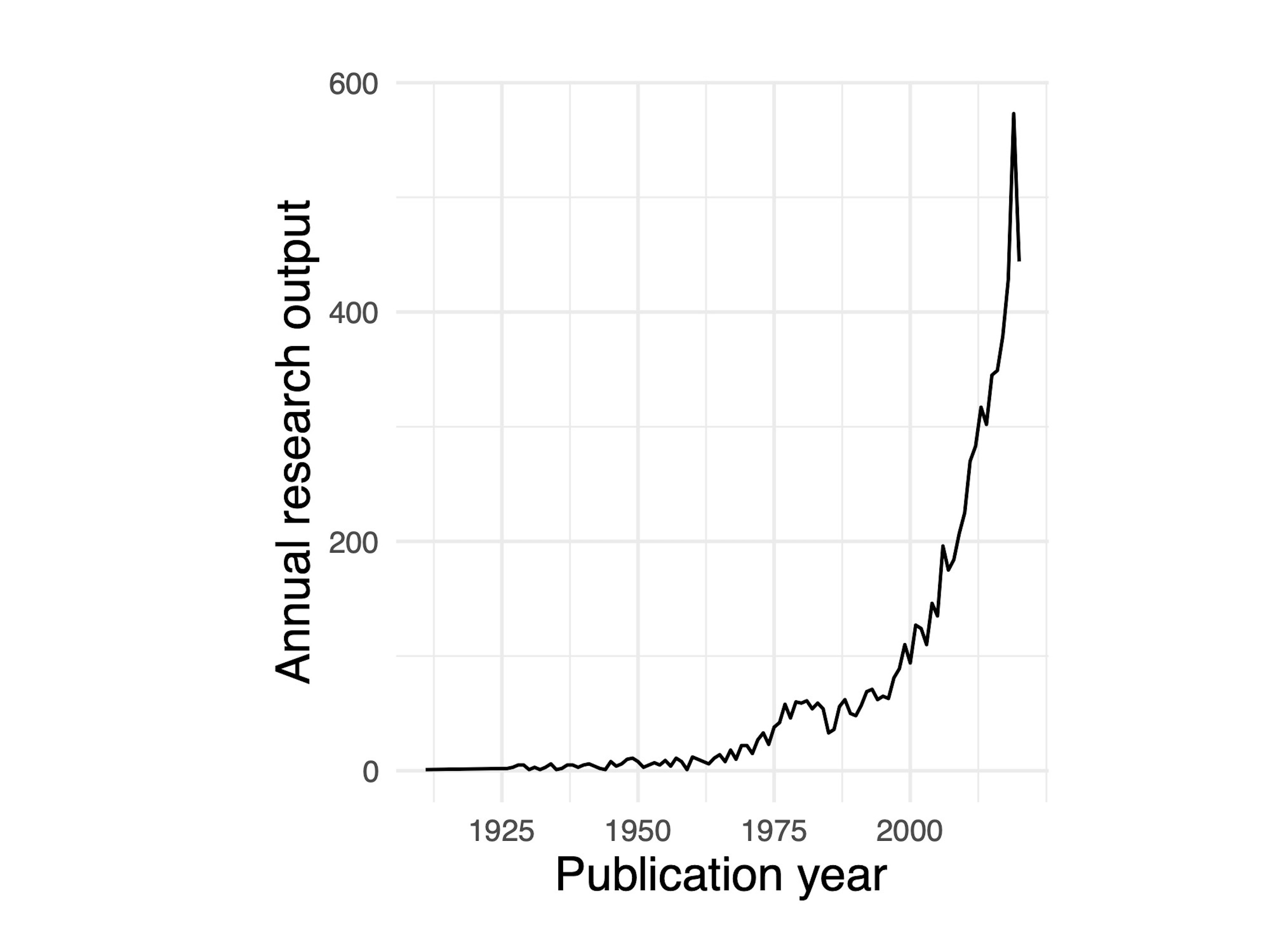

Supplement: Supplementary Figure 1 — Publication trend on fish skin microbiome. A Web of Science topic search [using the search string (skin OR epiderm*) AND (microb* OR bacteri* OR microorg* OR microflora OR microbiome OR microbiota) AND fish*] conducted on 5th of January 2021 yielded 7,299 publications indicating that scientific output on fish skin-associated microbiomes is limited but growing by each year. [file Image_1.JPEG]

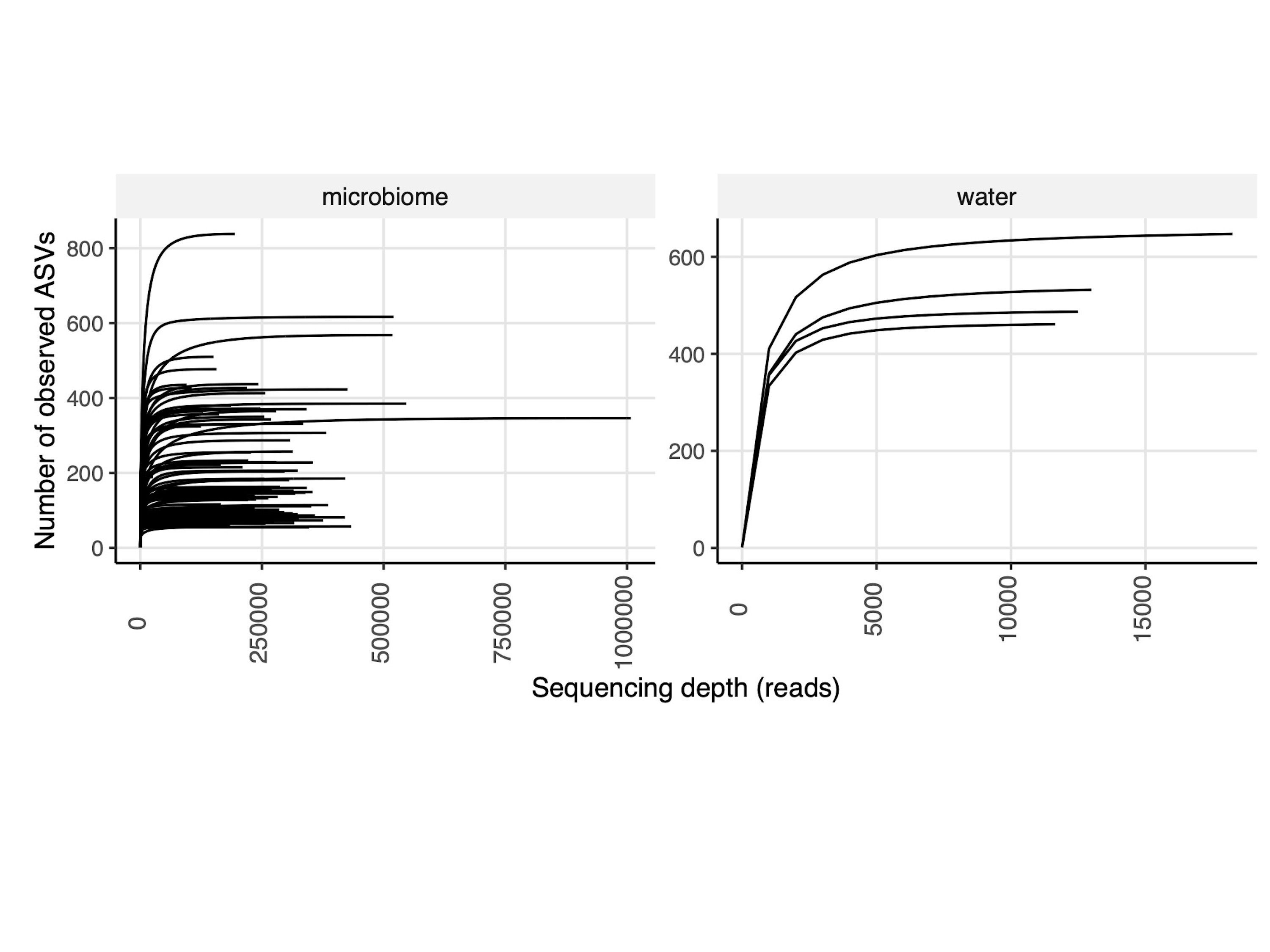

Supplement: Supplementary Figure 2 — Rarefaction curves for fish skin-associated microbiomes and bacterioplankton communities in water samples. Curves display a sufficient sequence coverage. [file Image_2.JPEG]
