# Supplementary material for: Fish Skin Microbiomes Are Highly Variable Among Individuals and Populations but Not Within Individuals
Source: Front Microbiol. 2022 Jan 21;12:767770. doi: 10.3389/fmicb.2021.767770 (PMC8813977; doi:10.3389/fmicb.2021.767770)
Supplement: Supplementary Data File 1 — ASV-table and taxonomy. [file Data_Sheet_1.PDF]

---
title: "Perch_MS"
author: "Hanna Berggren"
date: "12/17/2020"
output: document
editor_options:
  chunk_output_type: console

#Load required packages
```{r}
library(tidyverse)
library(dplyr)
library(tidyr)
library(vegan)
library(ggplot2)
library(ampvis2)
library(pastecs)
library(ggpubr)
library(car)
library(cowplot)
```

###
###############
################################ Pre - processing
###
###
###############
#ALPHA DIVERSITY

#Calculating estimated richness with breakaway package:

```{r}
library(breakaway)
```

```{r}
perch_counts <- read_tsv("perch_counts_filterd.tsv")
n_distinct(perch_counts$sample)
```

~~~
perch_counts %>% spread(sample, count, fill = 0) %>% data.frame() %>%
tibble::column_to_rownames('seqid') %>% breakaway() -> est_rich

est_rich_t <- tibble(
  sample = names(est_rich)
) %>%
  mutate(
    est = purrr::map(sample, function(s) est_rich[[s]]$estimate),
    error = purrr::map(sample, function(s) est_rich[[s]]$error),
    ci = purrr::map(sample, function(s) est_rich[[s]]$ci),
    ) %>%
  unnest(c(est, error, ci)) %>%
  group_by(sample) %>%
  mutate(cia = sprintf("ci%d", rank(ci))) %>%
  ungroup() %>%
  spread(cia, ci)


write_tsv(est_rich_t, "perch_richness_all.txt")

```
~~~

#BETA DIVERSITY

CLR -values

#Loading packages needed to calculate clr's

~~~
```{r libraries, message=F, cache = FALSE}

library(zCompositions)
library(CoDaSeq)
```
~~~

#For water and microbiome samples together:

~~~
```{r}
perch_water_counts <-read_tsv('perchwater_counts_P13155.tsv')

perch_counts <- read_tsv("perch_counts_filterd.tsv")

perch_counts <- dplyr::bind_rows(perch_counts, perch_water_counts)
```


```{r eval=FALSE, include=FALSE}
~~~

~~~~
# This *adds a clr column* to the asvs table.
# Note that the assignment to "asvs" is last and uses the -> assignment operator.
# Since all combinations of sample and seqid will now get a value, clr, the number of rows
# increases quite a lot.
perch_counts %>%
  dplyr::select(sample, count, seqid) %>%
  # Make the table wide with samples as columns
  spread(sample, count, fill = 0) %>%
  # Move the seqid to rowname; this requires a data.frame()
  data.frame() %>% tibble::column_to_rownames('seqid') %>%
  # Replace zeroes with probabilities (pseudocounts) (I needed a slightly lower delta than default not to get negative values)
  cmultRepl(method = 'CZM', delta = 0.5, output = 'p-counts') %>%
  # Calculate the CLR
  codaSeq.clr(samples.by.row = FALSE) %>%
  data.frame() %>%
  tibble::rownames_to_column('sample') %>%
  gather(seqid, clr, 2:ncol(.)) %>%
  # Get rid of 'X' that sometimes precedes the seqid and join back with original table
  mutate(seqid = sub('^X', '', seqid)) %>%
  left_join(perch_water_counts, by = c('sample', 'seqid')) %>%
  # Set count to 0 if it's NA
  replace_na(list(count = 0)) -> perch_counts_all_clr
```

# Save the dataframe with the clr values based on wtaer and microbiome together
```{r eval=FALSE, include=FALSE}
write_tsv(perch_counts_all_clr,"perch_counts_all_clr.tsv" )
```


# Transformed data such as clr-values and richness estimates are summarized in a common metadata file: perch_summary_metadata.tsv


# ########################################## END OF PRE_PROCESSING
############################


####################################################################################
###############
################################# Literature search
###########################################
####################################################################################
###############
~~~~

````
```{r}
data <- read_tsv("literature.txt")

data <- data %>% filter(!Years == "2021")

sum(data$records) #7299
```
#Figure S1 w=500 H=340
```{r}
p <- ggplot(data, aes(Years, records)) +
  #geom_point() +
  geom_line() +
  #xlim(1900, 2020)+
  #scale_color_manual(values = c("#DA70D6", "#800080", "#5F9EA0")) +
  scale_x_continuous(breaks = c(1900, 1925, 1950, 1975, 2000))+
  theme_minimal() +
  xlab("Publication year") +
  ylab("Annual research output") +

  #theme(legend.position = "none")  +
  theme(axis.title = element_text(size=14)) +
  theme(plot.title = element_text(size = 14))

p
```

#Figure S2 Rarefaction curves:

#Rarefaction curves, water and mucus:
```{r}

samples <- read_tsv("perch_summary_metadata.tsv", show_col_types = FALSE)

n_distinct(samples$sample) #114  samples: 110 microbiome, and 4 water samples

perch_water_counts <-read_tsv('perchwater_counts_P13155.tsv')
n_distinct(perch_water_counts$sample)#4

perch_counts <- read_tsv("perch_counts_filterd.tsv",  show_col_types = FALSE)

n_distinct(perch_counts$sample) #110

perch_counts <- dplyr::bind_rows(perch_counts, perch_water_counts)
```
#Taxonomy
```{r}
#Water taxonomy:
````
````

````
q2_tax_water <- read_tsv(
  'q2_perch_water_taxonomy.txt', col_types = cols(
  `Feature ID` = col_character(),
  Taxon = col_character(),
)) %>%
  mutate(
    Taxon = gsub('D_[0-9]_+', '', Taxon)
) %>%
  rename(seqid = `Feature ID`) %>%
  mutate(Taxon = gsub('[a-z]__', '', Taxon)) %>%
  separate(Taxon, c('domain', 'phylum', 'class', 'order', 'family', 'genus', 'species'), sep = ';', fill = 'right')

q2_tax_water = q2_tax_water[-1,] #remove first row with q2 specified types

n_distinct(q2_tax_water$seqid) #1278

#MUCUS TAXONOMY
taxonomy <- read_tsv("taxonomy_q2_filtered_20200409.tsv")

taxonomy <- taxonomy %>% semi_join(perch_counts, by = "seqid") #5778

n_distinct(taxonomy$seqid)

taxonomy <- bind_rows(taxonomy, q2_tax_water) %>% distinct(seqid, .keep_all = TRUE)

n_distinct(taxonomy$seqid) #7056 , this is water samples, plus the 110 mucus samples :-)
```
#Prepare data for ampviz
```{r}
#First, I need to convert the count frame to an OTU-table with sequences as rows and samples as column:
asv_table <- perch_counts %>% dplyr::select(sample, seqid, count) %>%
  spread(sample, count, fill = 0)

#Then I need to join this with the taxonomy based on seqid
asv_table <- asv_table %>%
  inner_join(taxonomy, by = 'seqid')

names(asv_table)[1] <- "OTU"

asv_table <- asv_table %>% dplyr::rename(Kingdom = domain, Phylum = phylum, Class = class, Order = order,  Family = family, Genus = genus, Species = species)
````

amp_data <- amp_load(asv_table, metadata = samples)

```
#Draw plot:Figure S2
```{r}
amp_rarecurve(amp_data, stepsize = 1000, color_by = NULL,
  facet_by = "sample_type", facet_scales = "free") +
  ylab("Number of observed ASVs")


#ggsave("FigureS2.eps", units = c("mm"), dpi = 300, height = 80, width = 180 )
```

##
################
################################## ALPHA DIVERSITY
##
##
################

Load Metadata

```{r}
samples <- read_tsv("perch_summary_metadata.tsv", show_col_types = FALSE)

samples$flowcell<-factor(samples$flowcell)
samples$body_site<-factor(samples$body_site)
samples$fish_nr<-factor(samples$fish_nr)
samples$pop<-factor(samples$pop)
n_distinct(samples$sample)#114
samples <- samples %>% filter( !sample_type == "water")
n_distinct(samples$fish_nr)#39
n_distinct(samples$sample)#110

```


#Figure 1
#Alpha diversity figure by AF
Preparing data for Richness-figure by AF
We need info about individ and
in addition, how many ASVs in total
# i)	in Kalmar
# ii)	in Figeholm
# iii)	and Kalmar + Figeholm
#Extract data for AF:
```{r}
perch_counts <- read_tsv("perch_counts_filtered_asvs.tsv")

```
taxonomy <- read_tsv("taxonomy_q2_filtered_20200409.tsv")

perch_counts <- perch_counts %>%
  inner_join(taxonomy, by = 'seqid')

perch_counts <- perch_counts %>%
  inner_join(samples, by = "sample")

#Number of ASVs per sample:
perch_counts <- perch_counts %>%
  group_by(sample) %>%
  mutate(AVSs_sample = n_distinct(ASV)) %>%
  ungroup()

#NUmber of ASVs found on each fish individual
perch_counts <- perch_counts %>%
  group_by(fish_ID) %>%
  mutate(number_of_ASVs = n_distinct(ASV)) %>%
  ungroup()

#NUmber of ASVs found on each fish individual
perch_counts <- perch_counts %>%
  group_by(fish_ID, body_site) %>%
  mutate(ASVs_individ_bodysite = n_distinct(ASV)) %>%
  ungroup()

#NUmber of ASVs found in each population
perch_counts <- perch_counts %>%
  group_by(pop) %>%
  mutate(ASVs_pop = n_distinct(ASV)) %>%
  ungroup()

perch_counts <- perch_counts %>%
  mutate(ASVs_total = n_distinct(ASV)) %>%
  ungroup()

t <- perch_counts %>% distinct(sample, .keep_all = TRUE)

#write_tsv(t, "alpha_beta_gamma.tsv")

```


# ANOVA

```{r}
perch_counts <- read_tsv("perch_counts_filterd.tsv", show_col_types = FALSE)

taxonomy <- read_tsv("taxonomy_q2_filtered_20200409.tsv", show_col_types = FALSE) #


perch_counts <- perch_counts %>%
  inner_join(taxonomy, by = 'seqid')

perch_counts <- perch_counts %>%
  inner_join(samples, by = "sample") %>% filter(!sample_type=="water")

#Number of ASVs found on each fish individual
perch_counts <- perch_counts %>%
  group_by(fish_ID) %>%
  mutate(number_of_ASVs = n_distinct(seqid)) %>%
  ungroup()
#Save only the unique fish indiviuals
t2 <- perch_counts %>% distinct(fish_ID, .keep_all = TRUE)

#One-way ANOVA:
number_samples <- aov(number_of_ASVs ~number_samples, data = t2)
summary(number_samples)

#
#              Df  Sum Sq Mean Sq F value Pr(>F)
#number_samples  1  131355  131355   1.773  0.191
#Residuals      37 2741460   74094
```

```{r}
perch_counts <- read_tsv("perch_counts_filterd.tsv", col_types =  cols(
  seqid = col_character(),
  sample = col_character(),
  count = col_double()
))

perch_counts <- perch_counts %>%
  inner_join(samples, by = 'sample') %>%
  dplyr::select(seqid, sample, count)

n_distinct(perch_counts$seqid) # 5778

taxonomy <- read_tsv("taxonomy_q2_filtered_20200409.tsv", col_types = cols(
```

  seqid = col_character(),
  domain = col_character(),
  phylum = col_character(),
  class = col_character(),
  order = col_character(),
  family = col_character(),
  genus = col_character(),
  species = col_character(),
))

n_distinct(taxonomy$phylum) #How many phyla? 57
unique(taxonomy$phylum)   #The names of the phyla

```

###########################################################################
########################
#################################      TAXONOMY
#############################################
###########################################################################
########################

# CORE MICROBIOME
```{r}
perch_counts <- read_tsv("perch_counts_filterd.tsv", show_col_types = FALSE)

perch_counts <- perch_counts %>%
  inner_join(samples, by = 'sample') %>%
  dplyr::select(seqid, sample, count)

taxonomy <- read_tsv("taxonomy_q2_filtered_20200409.tsv", col_types = cols(
  seqid = col_character(),
  domain = col_character(),
  phylum = col_character(),
  class = col_character(),
  order = col_character(),
  family = col_character(),
  genus = col_character(),
  species = col_character(),
))

n_distinct(taxonomy$phylum) #How many phyla? 57
unique(taxonomy$phylum)     #The names of the phyla

```
```

#The most shared ASVs, core microbiome. Here defined as present in at least 80% of the samples

```{r}
#All samples, 3 ASVs, All belong to Proteobacteria:
t <-  perch_counts %>%
  group_by(seqid) %>%
  summarise(sharedSamples = n(),
          perc_shared = sharedSamples/110) %>%
  ungroup()
#ASVs present in at least 80% of samples:
#aa35a552250ecd97c9c7b9c1f8359b52 #Shared among 106 samples Variovorax
#64190fa4ad2b9dbe1daa44b34f7b76fa #98 samples Burkholderiaceae
#56577f057b750e1902c2915852f122a1 #92 samples Rhizobialez

#Kalmar, 5 ASVs:
t2 <-  perch_counts %>% inner_join(samples, by="sample") %>% filter(pop=="Kalmar") %>%
  group_by(seqid) %>%
  summarise(sharedSamples = n(),
          perc_shared = sharedSamples/82) %>%
  ungroup()

#ASVs present in at least 80% of samples:
#56577f057b750e1902c2915852f122a1 #Shared among 65 samples Rhizobialez
#0b14daaaab979f25d205e686669fe844 #66 samples  Halomonas
#d2448b3d3ce0ee290d270f614ef96f7a #67 Aeromonas
#64190fa4ad2b9dbe1daa44b34f7b76fa #72 samples Burkholderiaceae
#aa35a552250ecd97c9c7b9c1f8359b52 #79 samplesVariovorax

#Figeholm, 3 ASVs:
t3 <-  perch_counts %>% inner_join(samples, by="sample") %>% filter(pop=="Figeholm")
%>%
  group_by(seqid) %>%
  summarise(sharedSamples = n(),
          perc_shared = sharedSamples/28) %>%
  ungroup()

#ASVs present in at least 80% of samples:
#64190fa4ad2b9dbe1daa44b34f7b76fa #Shared among 26 samples Burkholderiaceae
#aa35a552250ecd97c9c7b9c1f8359b52 #27  Variovorax
#56577f057b750e1902c2915852f122a1 #27  Rhizobialez


```

#DISTRIBUTION OF THE CORE PHYLA AMONG SAMPLES
#Total number of sequnces are:30, 308 550

```{r}
#Per 3 phyla present in all samples:
counts_Bacteriodetes <- perch_counts %>%
  inner_join(taxonomy, by = 'seqid') %>%
  filter(phylum=="Bacteroidetes")

  sum(counts_Bacteriodetes$count) #7011061/30, 308 550 = 23%  of total read abundance

counts_Actinobacteria <- perch_counts %>%
  inner_join(taxonomy, by = 'seqid') %>%
  filter(phylum=="Actinobacteria")

  sum(counts_Actinobacteria$count) #2374658/30, 308 550 = 7.8%  of total read abundance

counts_Proteobacteria <- perch_counts %>%
  inner_join(taxonomy, by = 'seqid') %>%
  filter(phylum=="Proteobacteria")

  sum(counts_Proteobacteria$count) #7902401/30, 308 550 = 26% of total read abundance

  #Mean and sd read abundance for samples of the core phyla:
#Actinobacteria meand: 0.09327991 +/- #0.18735447
#Bacteroidetes 0.24005681 +/- 0.19337313
#Proteobacteria mean: 0.25078634 +/- 0.13507720

  #SUMMARY
  #Actinobacteria mean among all samples 9.3% (range = 0.003-82%),
#Bacteroidia 0.23631382 0.19529324, 24% (range = 0.02-80%)
#Proteobacteria::Gammaproteobacteria 0.16346201 0.10232965, 25% (range = 2.7-55%)
```

#Figure 3: Boxplot on core phyla:

```{r}
samples <- read_tsv("perch_summary_metadata.tsv", show_col_types = FALSE)

samples$flowcell<-factor(samples$flowcell)
samples$body_site<-factor(samples$body_site)
samples$fish_nr<-factor(samples$fish_nr)
samples$pop<-factor(samples$pop)
n_distinct(samples$sample)#114
samples <- samples %>% filter( !sample_type == "water")
n_distinct(samples$fish_nr)#39
n_distinct(samples$sample)#110

perch_counts <- read_tsv("perch_counts_filterd.tsv",show_col_types = FALSE )
```

````
perch_counts <- perch_counts %>%
  group_by(sample) %>% mutate(relab = count/sum(count))
taxonomy <- read_tsv("taxonomy_q2_filtered_20200409.tsv", show_col_types = FALSE)
```

```{r}
perch_counts <- perch_counts %>%
  left_join(taxonomy, by = "seqid") %>% left_join(samples, by ="sample")

library(harrypotter)
hp(n = 7, house = "LunaLovegood")
# Use the following colors: #"#276C69FF" "#73C1C4FF" "#830042FF"

ggarrange(
perch_counts %>% filter(count >0) %>% filter(pop=="Kalmar") %>%
#inner_join(taxonomy, by = "seqid") %>%
  #group_by(phylum, pop)%>% summarise(relab =sum(relab_pop)) %>% ungroup()%>%
  filter(phylum  %in% c("Proteobacteria", "Actinobacteria" , "Bacteroidetes"))%>%
  group_by(sample, phylum)%>%
  summarise(relab= sum(relab))%>%
  ungroup()%>%
  ggplot(aes(x=phylum, y=relab)) +
  geom_boxplot(aes(fill=factor(phylum))) +
  #scale_fill_hp_d(option = "LunaLovegood")+
  #scale_color_manual(values = c("#008080", "#2F4F4F","#790149"))+
   scale_fill_manual(values = c("#276C69FF", "#73C1C4FF", "#830042FF" ))+
  #geom_violin(fill= NA, aes(color=phylum))+
  #geom_jitter(aes(color=phylum))+
  #geom_point(aes(color=phylum))+
  theme_classic()+
  theme(axis.text.x = element_blank())+
  theme(axis.text = element_text(size = 11))+
  theme(legend.position = "bottom")+
  theme(legend.title = element_blank())+
  theme(legend.text = element_text(size = 12))+
  ylim(0,1)+
  theme(axis.title = element_text(size=14))+
  xlab("Kalmar")+
  ylab("Relative abundance"),
perch_counts %>% filter(count >0) %>% filter(pop=="Figeholm") %>%
#inner_join(taxonomy, by = "seqid") %>%
  #group_by(phylum, pop)%>% summarise(relab =sum(relab_pop)) %>% ungroup()%>%
  filter(phylum  %in% c("Proteobacteria", "Actinobacteria" , "Bacteroidetes"))%>%
  group_by(sample, phylum)%>%
  summarise(relab= sum(relab))%>%
  ungroup()%>%
  ggplot(aes(x=phylum, y=relab)) +
````

````
  geom_boxplot(aes(fill=factor(phylum))) +
  #geom_jitter(aes(color=factor(phylum))) +
  #geom_boxplot(outlier.shape = NA)+
  #fill_color_manual(values = c("#008080", "#2F4F4F","#790149" ))+

scale_fill_manual(values = c("#276C69FF", "#73C1C4FF", "#830042FF" ))+
  #scale_fill_hp_d(option = "LunaLovegood")+
  #geom_violin(fill= NA, aes(color=phylum))+
  #geom_jitter(aes(color=phylum))+
  #geom_point(aes(color=phylum))+
  theme_classic()+
  theme(legend.position = "none")+
  theme(axis.text.x = element_blank())+
  theme(axis.text = element_text(size = 11))+
  ylim(0,1)+
  theme(axis.title = element_text(size=14))+
  xlab("Figeholm") +
  ylab(""), nrow=1, ncol = 2, common.legend =TRUE)

#ggsave("Figure3.eps", dpi = 300, width = 180, height = 95, units = c("mm")) for frontiers

```

#TAXONOMIC STACKED BARCHART

#Microbiome taxonomy:
```{r}
samples <- read_tsv("perch_summary_metadata.tsv", show_col_types = FALSE)

samples$flowcell<-factor(samples$flowcell)
samples$body_site<-factor(samples$body_site)
samples$fish_nr<-factor(samples$fish_nr)
samples$pop<-factor(samples$pop)
n_distinct(samples$sample)#114
samples <- samples %>% filter( !sample_type == "water")
n_distinct(samples$fish_nr)#39
n_distinct(samples$sample)#110

perch_counts <- read_tsv("perch_counts_filterd.tsv",show_col_types = FALSE )
perch_counts <- perch_counts %>%
  inner_join(samples, by = "sample")
taxonomy <- read_tsv("taxonomy_q2_filtered_20200409.tsv", show_col_types = FALSE)
```

```{r}
#  --------------------------------------------------
````

```
#The most abundant phyla is calculated per population.
#Moreover, since we want to to display individual bars later in in the figure, we calculate the relative abundance based on individuals already here:
#INDIVIDUAL
perch_counts <- perch_counts %>%
 group_by(fish_nr) %>%
 mutate(relab_ind = count/sum(count)) %>%
 ungroup()


#How many phyla in each pop?
kalmar <- perch_counts %>% filter(pop=="Kalmar") %>%
  left_join(taxonomy, by ="seqid")

n_distinct(kalmar$phylum) #52

figeholm <- perch_counts %>% filter(pop=="Figeholm") %>%
  left_join(taxonomy, by ="seqid")

n_distinct(figeholm$phylum) #47

kalmar <- kalmar %>% group_by(sample) %>%
 mutate(relab = count/sum(count)) %>%
 ungroup()

top12phyla_K <- kalmar %>%
  group_by(phylum, sample) %>%
  summarise(relab =sum(relab)) %>%
  summarise(meanrelab = mean(relab), sd = sd(relab),minrelab = min(relab), maxrelab = max(relab)) %>%
  ungroup() %>%
  top_n(12, meanrelab) %>% as.data.frame()

#Referee2 wants the order ascending:
top12phyla_K <- top12phyla_K[order(top12phyla_K$meanrelab, decreasing =  FALSE),]

top12phyla_K %>% distinct(phylum)
#                       phylum
#1               Planctomycetes
#2               Verrucomicrobia
#3                 Crenarchaeota
#4           Deinococcus-Thermus
#5                   Tenericutes
#6                   Chloroflexi
#7  uncultured archaeon 'KTK 28A'
#8              Gemmatimonadetes
#9                 Euryarchaeota
```

```
#10            Actinobacteria
#11            Proteobacteria
#12             Bacteroidetes

#I need to order the shared phyla in the levels for increasing ABUNDANCE IN FIGURE 4 for
each top12
top12phyla_K$phylum <- factor(top12phyla_K$phylum, levels = c( "Other phyla"
,"Bacteroidetes",  "Proteobacteria", "Actinobacteria", "Euryarchaeota",
"Gemmatimonadetes", "uncultured archaeon 'KTK 28A'", "Chloroflexi","Tenericutes",
"Deinococcus-Thermus","Crenarchaeota",  "Verrucomicrobia",  "Planctomycetes" ))


top12phyla_K$phylum
#Prevalence of phyla among samples in Kalmar
tk <- kalmar %>% select(sample, seqid) %>%
  left_join(taxonomy %>% select(phylum, seqid)) %>%
  select(-seqid) %>%
  unique() %>%
  group_by(phylum) %>%
  summarise(shared_samples = n(),
          percentage_shared = shared_samples/82)
#Actinobacteria, Bacteroidetes, Proteobacteria, Patescibacteria is present among all
samples

prevalence_phyla_K <- kalmar %>%
  group_by(phylum, sample) %>%
  summarise(relab =sum(relab)) %>%
  summarise(meanrelab = mean(relab), sd = sd(relab),minrelab = min(relab), maxrelab =
max(relab)) %>%
  ungroup()

prevalence_phyla_K$sample_type <- "microbiome"
prevalence_phyla_K$pop <- "Kalmar"


#Figeholm:
figeholm <- figeholm %>%group_by(sample) %>%
 mutate(relab = count/sum(count)) %>%
 ungroup()
#Prevalence of phyla among samples in Figeholm
tf <- figeholm %>% select(sample, seqid) %>%
  left_join(taxonomy %>% select(phylum, seqid)) %>%
  select(-seqid) %>%
  unique() %>%
  group_by(phylum) %>%
  summarise(shared_samples = n(),
          percentaged_shared = shared_samples/28)
```

```
#Actinobacteria, Bacteroidetes and Proteobacteria are present among all samples

prevalence_phyla_F <- figeholm %>%
  group_by(phylum, sample) %>%
  summarise(relab =sum(relab)) %>%
  summarise(meanrelab = mean(relab), sd = sd(relab),minrelab = min(relab), maxrelab =
max(relab)) %>%
  ungroup()

prevalence_phyla_F$sample_type <- "microbiome"
prevalence_phyla_F$pop <- "Figeholm"


top12phyla_F <- figeholm %>%
  group_by(phylum, sample) %>%
  summarise(relab =sum(relab)) %>%
  summarise(meanrelab = mean(relab), sd = sd(relab),minrelab = min(relab), maxrelab =
max(relab)) %>%
  ungroup() %>%
  top_n(12, meanrelab) %>% as.data.frame()

#How to calculate how many of the reads that are represented?

str(top12phyla_F)

top12phyla_F <- top12phyla_F[order(top12phyla_F$meanrelab, decreasing =  FALSE),]

top12phyla_F %>% distinct(phylum)

#NEW ORDER _ ASCENDING
#             phylum
#1       Cyanobacteria
#2  Deinococcus-Thermus
#3        Spirochaetes
#4      Actinobacteria
#5         Chloroflexi
#6           Aquificae
#7    Gemmatimonadetes
#8       Euryarchaeota
#9     Patescibacteria
#10      Bacteroidetes
#11     Proteobacteria
#12        Tenericutes


top12phyla_F$phylum <- factor(top12phyla_F$phylum, levels = c("Other phyla"
,"Tenericutes", "Proteobacteria","Bacteroidetes", "Patescibacteria", "Euryarchaeota",
```

````
"Gemmatimonadetes", "Aquificae", "Chloroflexi", "Actinobacteria",  "Spirochaetes",
"Deinococcus-Thermus",  "Cyanobacteria"))

top12phyla_F$phylum

```


#Water taxonomy:
```{r}
samples <- read_tsv("perch_summary_metadata.tsv", show_col_types = FALSE)

samples$flowcell<-factor(samples$flowcell)
samples$body_site<-factor(samples$body_site)
samples$fish_nr<-factor(samples$fish_nr)
samples$pop<-factor(samples$pop)
n_distinct(samples$sample)#114
samples <- samples %>% filter( !sample_type == "microbiome")
n_distinct(samples$fish_nr)#39
n_distinct(samples$sample)#110


perch_water_counts <-read_tsv('perchwater_counts_P13155.tsv')

perch_water_counts <- perch_water_counts %>% left_join(samples, by= "sample")

perch_water_counts <- perch_water_counts %>%
  group_by(pop) %>%  #needs to be pop otherwise the relative abundance scale will be 1 or
figeholm and 3 for Kalmar
  mutate(relab = count/sum(count)) %>%
  ungroup()

#Water taxonomy:

q2_tax_water <- read_tsv(
  'q2_perch_water_taxonomy.txt', col_types = cols(
  `Feature ID` = col_character(),
  Taxon = col_character()
)) %>%
  mutate(
    Taxon = gsub('D_[0-9]_+', '', Taxon)
  ) %>%
  rename(seqid = `Feature ID`) %>%
  mutate(Taxon = gsub('[a-z]__', '', Taxon)) %>%
  separate(Taxon, c('domain', 'phylum', 'class', 'order', 'family', 'genus', 'species'), sep = ';', fill
= 'right')
````

```
q2_tax_water = q2_tax_water[-1,] #remove first row with q2 specified types


n_distinct(q2_tax_water$seqid) #1278

top12phyla_water <- perch_water_counts %>%
  inner_join(q2_tax_water, by = 'seqid') %>%
  group_by(phylum, sample)%>%
  summarise(relab =sum(relab)) %>%
  summarise(meanrelab = mean(relab), sd = sd(relab), minrelab = min(relab), maxrelab =
max(relab)) %>%
  ungroup() %>%
  top_n(12, meanrelab)

top12phyla_water <- top12phyla_water[order(top12phyla_water$meanrelab, decreasing =
FALSE),]

unique(top12phyla_water$phylum)
#NEW ORDER
# [1] "Tenericutes"     "Dependentiae"    "Fusobacteria"    "Acidobacteria"   "Planctomycetes"
"Chloroflexi"
# [7] "Firmicutes"      "Verrucomicrobia" "Cyanobacteria"   "Proteobacteria"  "Bacteroidetes"
"Actinobacteria"

#top12phyla_water$phylum <- factor(top12phyla_water$phylum, levels = c("X Other
phylum", "Tenericutes" , "Dependentiae" , "Fusobacteria", "Acidobacteria" ,
"Planctomycetes" ,"Chloroflexi", "Firmicutes" , "Verrucomicrobia", "Cyanobacteria",
"Proteobacteria","Bacteroidetes",  "Actinobacteria"  ))

#The order need to be "reversed" if we want increasing abundance in Figure 4:
top12phyla_water$phylum <- factor(top12phyla_water$phylum, levels = c("Other phyla"
,"Actinobacteria"  , "Bacteroidetes", "Proteobacteria","Cyanobacteria","Verrucomicrobia",
"Firmicutes" ,  "Chloroflexi","Planctomycetes" , "Acidobacteria" ,"Fusobacteria",
"Dependentiae" , "Tenericutes" ))

prevalence_phyla_water <- perch_water_counts %>%
  inner_join(q2_tax_water, by = 'seqid') %>%
  group_by(phylum, sample) %>%
  summarise(relab =sum(relab)) %>%
  summarise(meanrelab = mean(relab), sd = sd(relab),minrelab = min(relab), maxrelab =
max(relab)) %>%
  ungroup()

prevalence_phyla_water$sample_type<- "water"
prevalence_phyla_water$pop<- "combined"

#Combine the prevalence daataframe for supplementaries:
```

~~~
prevalence_all <- rbind(prevalence_phyla_F, prevalence_phyla_K, prevalence_phyla_water)
#write_tsv(prevalence_all, "Phylum_prevalence_in_each_sample_type.tsv")

#Investigating what taxonomic groups that dominates the most abundant phyla:
   counts_actino <- perch_water_counts %>%
  inner_join(taxonomy, by = 'seqid') %>%
  filter(phylum=="Actinobacteria")

   Actino_abundance  <- counts_actino %>%
  group_by(family, sample) %>%
  summarise(relab =sum(relab)) %>%
  summarise(meanrelab = mean(relab), sd = sd(relab), minrelab = min(relab), maxrelab =
max(relab)) %>%
  ungroup() # Microbacteriaceae, Sporichthyaceae

   counts_bacter <- perch_water_counts %>%
  inner_join(taxonomy, by = 'seqid') %>%
  filter(phylum=="Bacteroidetes")

   Bacter_abundance  <- counts_bacter %>%
  group_by(family, sample) %>%
  summarise(relab =sum(relab)) %>%
  summarise(meanrelab = mean(relab), sd = sd(relab), minrelab = min(relab), maxrelab =
max(relab)) %>%
  ungroup() # Flavobacteriaceae 6.7 +/- 4.7%

    counts_prot <- perch_water_counts %>%
  inner_join(taxonomy, by = 'seqid') %>%
  filter(phylum=="Proteobacteria")

   Prot_abundance  <- counts_prot %>%
  group_by(family, sample) %>%
  summarise(relab =sum(relab)) %>%
  summarise(meanrelab = mean(relab), sd = sd(relab), minrelab = min(relab), maxrelab =
max(relab)) %>%
  ungroup() # Burkholderiacea, Rhodobacteriaceae

```


#Figure 4: Stacked barchart
```{r }
ggarrange(perch_counts %>% filter(pop=="Kalmar") %>%
  # Join in the taxonomy, to get access to the phylum for each ASV
  inner_join(taxonomy, by = 'seqid') %>%
  left_join(top12phyla_K %>% transmute(phylum, topph = phylum), by = 'phylum') %>%
  replace_na(list('topph' = 'Other phyla')) %>%
~~~

```
 # left_join(samples, by = "sample") %>%
  # Calculate a sum of relative abundance per sample and phylum
  group_by(topph, fish_nr) %>%
  summarise(relab_ind = sum(relab_ind)) %>%
  ungroup() %>%
  # Plot this sum on the y axis, with samples on the x. Use fill to show phyla.
  ggplot(aes(x = fish_nr, y = relab_ind, fill = topph)) +
  # geom_col for barplots from numerical data
  geom_col() +
  #scale_fill_hp_d(option = "LunaLovegood")+
   scale_fill_manual(values=c("#FDF5E6",  "#73C1C4FF","#008080", "#2E8B57","#663300",
"#DEB887", "#ADD8E6","#790149", "#FFCFE2" , "#FF9DC8", "#FFEBCD", "#A64264FF",
"#BF8699FF" ))+
   #scale_fill_brewer(palette = "BrBG")+
  # Flip the axes so you can read the sample names
  coord_flip() +
  theme(legend.position = "bottom")+
  theme(legend.title = element_blank()) +
  theme(legend.text = element_text(size=14))+
  theme(axis.text = element_text(size = 14))+
  theme(axis.title = element_text(size=14))+
  xlab("") +
  ylab("Relative abundance")+
  ggtitle(~bold("(A)") ~"Kalmar, individuals"),
perch_counts %>% filter(pop=="Figeholm") %>%
  # Join in the taxonomy, to get access to the phylum for each ASV
  inner_join(taxonomy, by = 'seqid') %>%
  left_join(top12phyla_F %>% transmute(phylum, topph = phylum), by = 'phylum') %>%
  replace_na(list('topph' = 'Other phyla')) %>%
 # left_join(samples, by = "sample") %>%
  # Calculate a sum of relative abundance per sample and phylum
  group_by(fish_nr, topph) %>%
  summarise(relab_ind = sum(relab_ind)) %>%
  ungroup() %>%
  # Plot this sum on the y axis, with samples on the x. Use fill to show phyla.
  ggplot(aes(x = fish_nr, y = relab_ind, fill = topph)) +
  # geom_col for barplots from numerical data
  geom_col() +
  scale_fill_manual(values=c( "#FDF5E6", "#FFCFE2" , "#008080","#73C1C4FF", "#FFF0F5",
"#663300", "#DEB887",   "#BC8F8F", "#790149","#2E8B57",  "#66CDAA",
"#FF9DC8","#AFEEEE"))+
  #scale_fill_brewer(palette = "BrBG")+
  #scale_fill_hp_d(option = "LunaLovegood")+
  # Flip the axes so you can read the sample names
  coord_flip() +
  theme(legend.position = "bottom")+
  theme(legend.title = element_blank()) +
```

````
  theme(legend.text = element_text(size=14))+
  theme(axis.text = element_text(size = 14))+
  theme(axis.title = element_text(size=14))+
  xlab("") +
  ylab("Relative abundance")+
  ggtitle(~bold("(B)")~ "Figeholm, individuals"),
perch_water_counts %>%
  #filter(pop=="Kalmar") %>%
  # Join in the taxonomy, to get access to the phylum for each ASV
  inner_join(q2_tax_water, by = 'seqid') %>%
  #Join in the metadata to get pop info:
  left_join(top12phyla_water %>% transmute(phylum, topph = phylum), by = 'phylum') %>%
  # Replace missing phyla with 'Unknown phylum'
  replace_na(list('topph' = 'Other phyla')) %>%
  # Calculate a sum of relative abundance per sample and phylum
  group_by(pop, topph) %>% summarise(relab = sum(relab)) %>% ungroup() %>%
  # Plot this sum on the y axis, with samples on the x. Use fill to show phyla.
  ggplot(aes(x = pop, y = relab, fill = topph)) +
  # geom_col for barplots from numerical data
  geom_col() +
  scale_fill_manual(values=c("#FDF5E6", "#2E8B57","#73C1C4FF","#008080",       "#AFEEEE"
,"#A64264FF",  "#66CDAA","#790149", "#BF8699FF" ,"#5F9EA0", "#C7007C",   "#7FFFD4",
"#FFCFE2" ))+
  theme(legend.position = "bottom")+
  theme(legend.title = element_blank()) +
  theme(legend.text = element_text(size=14))+
  theme(axis.text = element_text(size = 14))+
  theme(axis.title = element_text(size=14))+
  xlab("") +
  ylab("Relative abundance")+
  ggtitle(~bold ("(C)") ~"Water") +
  # Flip the axes so you can read the sample names
  coord_flip(),
  ncol = 1, nrow =4, heights = c(2.2,1.1,0.7), align = "v")

#size 1200*1500 Inserted in MS 2021 02 16
#ggsave("Figure4.eps", dpi = 600, units = c("mm"), width = 400, height = 600 )
```

##############################################################################
###############
################################## BETA DIVERSITY
###########################################
##############################################################################
###############
#Compare community composition in water and microbiomes:
```{r}
````

```
samples <- read_tsv("perch_summary_metadata.tsv")

samples$flowcell<-factor(samples$flowcell)
samples$body_site<-factor(samples$body_site)
samples$fish_ID<-factor(samples$fish_ID)
samples$pop<-factor(samples$pop)
samples$sample_type <-factor(samples$sample_type)
samples$fish_nr <- as.factor(samples$fish_nr)

n_distinct(samples$fish_nr)
n_distinct(samples$sample)


perch_counts_clr <- read_tsv('perch_counts_all_clr.tsv', col_types = cols(
  seqid = col_character(),
  sample = col_character(),
  count = col_double()
))

perch_counts_clr <- perch_counts_clr %>% semi_join(samples, by = "sample")

n_distinct(perch_counts_clr$sample)


# Create a matrix object; we need it named, can't generate one on "the fly"
perch_counts_clr %>% dplyr::select(sample, seqid, clr) %>%
  spread(seqid, clr) %>% tibble::column_to_rownames('sample') -> matrix_all

# Here's the call to the rda function with a formula as the first argument,
vegan::rda(
  matrix_all ~ sample_type , data = samples
) -> rda_all

anova.cca(rda_all, by="margin")
#Permutation test for rda under NA model
##Marginal effects of terms
#Permutation: free
#Number of permutations: 999
#
#Model: rda(formula = matrix_all ~ sample_type, data = samples)
#             Df Variance      F Pr(>F)
#sample_type   1   1598.7 17.744  0.001 ***
#Residual    112  10091.2
#---
head(summary(rda_all))
# adjusted R^2
```

~~~
R2adj_rda <- RsquareAdj(rda_all)$adj.r.squared
R2adj_rda
#0.129

```

########################################################################################################################
     3.1 Microbiome composition varied among host individuals from different source populations
########################################################################################################################

# RDA ANALYSIS ON MICROBIOME DATA#
#Load sample data
```{r}
samples <- read_tsv("perch_summary_metadata.tsv", show_col_types = FALSE)

samples$flowcell<-factor(samples$flowcell)
samples$body_site<-factor(samples$body_site)
samples$fish_ID<-factor(samples$fish_ID)
samples$fish_nr <- as.factor(samples$fish_nr)
samples$pop<-factor(samples$pop)
samples <- samples %>% filter(sample_type=="microbiome")

n_distinct(samples$fish_nr)
n_distinct(samples$sample)
```
#Load clr values for RDA
```{r}
perch_counts_clr <- read_tsv('perch_counts_clr.tsv', col_types = cols(
  seqid = col_character(),
  sample = col_character(),
  count = col_double()
))

#Join in with samples to get the correct samples for the clr values
perch_counts_clr <- perch_counts_clr %>%
  inner_join(samples, by = 'sample') %>%
  dplyr::select(sample, seqid, count, clr)
```

# Population specific effects
```{r calc-rda overall effects}
# Create a matrix object
perch_counts_clr %>% dplyr::select(sample, seqid, clr) %>%
  spread(seqid, clr) %>% tibble::column_to_rownames('sample') -> mucus_matrix
~~~

```
# Call to the rda function with a formula as the first argument,
vegan::rda(
  mucus_matrix ~ pop , data = samples
) -> rda
#Beacuse of the potential effect of flowcells, we need to restrict permutations:
how <- how(nperm=1000, plots = Plots(strata=samples$flowcell)) #restrict permutations to given groups;the two different flowcells P1295 and P1314

anova.cca(rda, by="margin", permu = how)
#Permutation test for rda under NA model
#Marginal effects of terms
#Permutation: free
#Number of permutations: 999

#Model: rda(formula = mucus_matrix ~ pop, data = samples)
#         Df Variance      F Pr(>F)
#pop       1    170.3 1.8024  0.006 **
#Residual 108  10206.9
#---
#Signif. codes:  0 ‘***’ 0.001 ‘**’ 0.01 ‘*’ 0.05 ‘.’ 0.1 ‘ ’ 1
head(summary(rda))
#Call:
#rda(formula = mucus_matrix ~ pop, data = samples)

##Partitioning of variance:
#             Inertia Proportion
#Total        10377.3    1.00000
#Constrained    170.3    0.01641
#Unconstrained 10206.9    0.98359

#There is an effect of population, however, there is only 1% of variance explained, compared to when individual is included, see below: 48%


# adjusted R^2, inserted in manuscript
R2adj_rda <- RsquareAdj(rda)$adj.r.squared
R2adj_rda
#0.007 for population



#Individual specific effects:
vegan::rda(
  mucus_matrix ~ fish_nr , data = samples
) -> rda
```

~~~
anova.cca(rda, by="margin", permu = how)
#Permutation test for rda under NA model
#Marginal effects of terms
#Permutation: free
#Number of permutations: 999

#Model: rda(formula = mucus_matrix ~ fish_nr, data = samples)
#        Df Variance      F Pr(>F)
#fish_nr  38   5036.4 1.7619  0.001 ***
#Residual 71   5340.8
#---
#Signif. codes:  0 ‘***’ 0.001 ‘**’ 0.01 ‘*’ 0.05 ‘.’ 0.1 ‘ ’ 1

#$adj.r.squared
# 0.209879 # for individual


head(summary(rda))
#Call:
#rda(formula = mucus_matrix ~ fish_nr, data = samples)

#Partitioning of variance:
#              Inertia Proportion
#Total          10377     1.0000
#Constrained      5036     0.4853
#Unconstrained    5341     0.5147


```

# PCA plot:

```{r mucus_pca plot}
pca.samples <- rda$CA$u %>% data.frame() %>% tibble::rownames_to_column('sample')
pca.asvs    <- rda$CA$v %>% data.frame() %>% tibble::rownames_to_column('asv')
pca.eigs    <- rda$CA$eig %>% data.frame() %>% tibble::rownames_to_column('pc') %>%
  rename(eigval = 2) %>%
  mutate(propexpl = eigval/sum(eigval))

# We use the pca.samples table as the "main" table when calling ggplot.
# Let's first join it with the samples table so we can use some metadata
# for colouring.
pca_plot <- pca.samples %>%
  inner_join(samples, by = 'sample') %>%
  ggplot(aes(x = PC1, y = PC2, color = fish_nr, shape = pop)) +

  # Points for samples, coloured by individual
~~~

~~~~
  geom_point(size = 3.5) +
  #stat_ellipse(aes(color=pop))+
  scale_size(guide = "none")+
  theme_classic() +
  theme(legend.position = "none") +

# increase text sizes
  theme(axis.title = element_text(size=15)) +
  theme(axis.text.x = (element_text(size = 10)))+
  theme(axis.text.y = (element_text(size = 10)))+
  xlab(sprintf("PC1 (%2.1f%% explained)", pca.eigs[1,3] * 100)) +
  ylab(sprintf("PC2 (%2.1f%% explained)", pca.eigs[2,3] * 100))

pca_plot

```




# BETA DISPERSION #
#Population level
# Make Euclidean matrix for betadsipersion analyses
```{r }
perch_euclidean <- perch_counts_clr %>%
  dplyr::select(sample, seqid, clr) %>%
  # We need a wide table with clr values, asvs as columns and samples as rows
  spread(seqid, clr) %>%
  tibble::column_to_rownames('sample') %>%
  # The dist functions calculates, by default, euclidian distances between rows in the table
  dist()%>%
  as.matrix()
```



```{r}
betadisp_eu <- betadisper(as.dist(perch_euclidean), samples$pop, type = "centroid",
bias.adjust = FALSE, sqrt.dist = FALSE, add = FALSE)
betadisp_eu
 "boxplot"(betadisp_eu, ylab = "Distance to centroid", xlab = "Population")

tukey<- "TukeyHSD"(betadisp_eu, ordered = FALSE, conf.level = 0.95)
anova_dispersion_pop <- anova(betadisp_eu)
distance_to_centroid_pop <- betadisp_eu$distances

samples <- samples %>% mutate(distance_to_centroid_pop= distance_to_centroid_pop)
~~~~

betadisp_eu$distances

#write_tsv(samples, "distance_pops.tsv")
```

#Individual effect in Kalmar
```{r}
perch_counts_clr %>% inner_join(samples_pop1, by = "sample") %>% dplyr::select(sample, seqid, clr) %>%
  spread(seqid, clr) %>% tibble::column_to_rownames('sample') -> pop1_matrix
vegan::rda(
  pop1_matrix ~  fish_ID,  data = samples_pop1) -> pop1_rda
how <- how(nperm=1000, plots = Plots(strata=samples$flowcell)) #restrict permutations to given groups;the two flowcells
anova(pop1_rda, by="margin", permu = how)

#Permutation test for rda under NA model
#Marginal effects of terms
#Permutation: free
#Number of permutations: 999

#Model: rda(formula = pop1_matrix ~ fish_ID, data = samples_pop1)
#        Df Variance      F Pr(>F)
#fish_ID  29   4825.9 1.6508  0.001 ***
#Residual 52   5241.8
#---
#Signif. codes:  0 ‘***’ 0.001 ‘**’ 0.01 ‘*’ 0.05 ‘.’ 0.1 ‘ ’ 1

head(summary(pop1_rda))
#Call:
#rda(formula = pop1_matrix ~ fish_ID, data = samples_pop1)

#Partitioning of variance:
#             Inertia Proportion
#Total          10068     1.0000
#Constrained      4826     0.4793
#Unconstrained    5242     0.5207

R2_rda_pop1 <- RsquareAdj(pop1_rda)$r.squared
R2_rda_pop1

#[1]0.4793445

# adjusted R^2
R2adj_rda_pop1 <- RsquareAdj(pop1_rda)$adj.r.squared

R2adj_rda_pop1

#0.1889789
```

#Test for individual in Figeholm
```{r}
pop2 <- samples %>% filter(!pop == "Kalmar") #All of these were sampled in Figeholm during 16/10 and sequenced on flowcell 1 (P1295)

n_distinct(pop2$fish_ID)
```

```{r}
perch_counts_clr %>% inner_join(pop2, by = "sample") %>% dplyr::select(sample, seqid, clr) %>%
  spread(seqid, clr) %>% tibble::column_to_rownames('sample') -> pop2_matrix
vegan::rda(
  pop2_matrix ~  fish_ID,  data = pop2) -> pop2_rda
#how <- how(nperm=100, plots = Plots(strata=pop2$flowcell)) #restrict permutations to given groups;not needed here
anova.cca(pop2_rda, by="margin")
#Permutation test for rda under NA model
#Marginal effects of terms
#Permutation: free
#Number of permutations: 999

#Model: rda(formula = pop2_matrix ~ fish_ID, data = pop2)
#         Df Variance      F Pr(>F)
#fish_ID   8   5167.0 2.1028  0.001 ***
#Residual 19   5835.8
#---
#Signif. codes:  0 ‘***’ 0.001 ‘**’ 0.01 ‘*’ 0.05 ‘.’ 0.1 ‘ ’ 1

head(summary(pop2_rda))
#Call:
#rda(formula = pop2_matrix ~ fish_ID, data = pop2)

#Partitioning of variance:
#              Inertia Proportion
#Total           11003     1.0000
#Constrained      5167     0.4696
#Unconstrained    5836     0.5304

# adjusted R^2
R2adj_rda_pop2 <- RsquareAdj(pop2_rda)$adj.r.squared
R2adj_rda_pop2

#0.246

```

###
########################
  3.2 Evaluating heterogeneity in microbiome community composition within individuals
###
########################
#Intraclass correlation coefficient on Estimated species richness
Load Metadata

```{r}
samples <- read_tsv("perch_summary_metadata.tsv", show_col_types = FALSE)

samples$flowcell<-factor(samples$flowcell)
samples$body_site<-factor(samples$body_site)
samples$fish_nr<-factor(samples$fish_nr)
samples$pop<-factor(samples$pop)
n_distinct(samples$sample)#114
samples <- samples %>% filter( !sample_type == "water")
n_distinct(samples$fish_nr)#39
n_distinct(samples$sample)#110

```

#For ICC analysis to filter out the individuals with 4 samples:
```{r}
samples_site <- samples %>% filter(!fish_nr == "2", !fish_nr == "3",!fish_nr == "4", !fish_nr == "5", !fish_nr == "6", !fish_nr == "7", !fish_nr == "8", !fish_nr == "9", !fish_nr == "10", !fish_nr == "12", !fish_nr == "14", !fish_nr == "15",  !fish_nr == "18", !fish_nr == "19", !fish_nr == "20", !fish_nr == "22", !fish_nr == "23", !fish_nr == "24", !fish_nr == "27", !fish_nr == "29", !fish_nr == "32", !fish_nr == "36", !fish_nr == "37", !fish_nr == "39", !fish_nr == "40", !fish_nr == "41", !fish_nr == "42", !fish_nr == "44", !fish_nr == "45")

n_distinct(samples_site$fish_nr)

library(psych)

```

#Dorsal
```{r}
icc_dorsal_est <- read_tsv("icc_dorsal_est.txt")

icc_dorsal_est <-icc_dorsal_est %>% dplyr::select( DL_est, DR_est)

dorsal_icc <- ICC(icc_dorsal_est, lmer = FALSE)
dorsal_icc

#Single_raters_absolute:     ICC1=  0.8556462     F= 12.85485   df1=15 df2=16
     p=0.000003336781   0.6435939     0.946605
```
#Ventral
```{r}
icc_ventral_est <- read_tsv("icc_ventral_est.txt")

icc_ventral_est <-icc_ventral_est  %>% dplyr::select( VL_est, VR_est)

ventral_icc <- ICC(icc_ventral_est, lmer = FALSE)
ventral_icc

#Single_raters_absolute:     ICC1=  0.8707012     F= 14.46805   df1=15 df2=16
     p=0.0000014575554  0.6769139     #0.9524173
```

#Dispersion plots for individual should be based on the 16 individuals with 4 samples, as the analysis in Primer7:

```{r}
samples_site <- samples %>% filter(!fish_nr == "2", !fish_nr == "3",!fish_nr == "4", !fish_nr == "5", !fish_nr == "6", !fish_nr == "7", !fish_nr == "8", !fish_nr == "10", !fish_nr == "12", !fish_nr == "14", !fish_nr == "15",  !fish_nr == "18", !fish_nr == "19",  !fish_nr == "20", !fish_nr == "22", !fish_nr == "23", !fish_nr == "24", !fish_nr == "27", !fish_nr == "29", !fish_nr == "32", !fish_nr == "36", !fish_nr == "37", !fish_nr == "39", !fish_nr == "40", !fish_nr == "41", !fish_nr == "42", !fish_nr == "44", !fish_nr == "45")

n_distinct(samples_site$fish_nr
     )
```


```{r}
perch_counts_clr <- read_tsv('perch_counts_clr.tsv', col_types = cols(
  seqid = col_character(),
  sample = col_character(),
  count = col_double()
))

perch_counts_clr <- perch_counts_clr %>%
  inner_join(samples_site, by = 'sample') %>%
  dplyr::select(sample, seqid, count, clr)
```
#Make Euclidean matrix for dispersion analyses of individuals
```

````
```{r }
perch_euclidean_ind <- perch_counts_clr %>%
  dplyr::select(sample, seqid, clr) %>%
  # We need a wide table with clr values, asvs as columns and samples as rows
  spread(seqid, clr) %>%
  tibble::column_to_rownames('sample') %>%
  # The dist functions calculates, by default, euclidian distances between rows in the table
  dist()%>%
  as.matrix()
```


#Figure 5
```{r}

#for each individual- within individual comparison:
betadisp_ind <- betadisper(as.dist(perch_euclidean_ind), samples_site$fish_ID, type =
"centroid", bias.adjust = FALSE, sqrt.dist = FALSE, add = FALSE)
"boxplot"(betadisp_ind, ylab = "Distance to centroid", xlab = "Fish ID", color =
samples_site$fish_ID)

anova_dispersion_individ <- anova(betadisp_ind)

distance_to_centroid_individ <- betadisp_ind$distances

within <- samples_site%>% mutate(distance = distance_to_centroid_individ)
%>%dplyr::select(distance)
within$distance_type <- "within"

#For Figure 5: distance based on all samples i.e., between individuals comparisons
  betadisp_all <- betadisper(as.dist(perch_euclidean_ind), samples_site$sample_type, type =
"centroid", bias.adjust = FALSE, sqrt.dist = FALSE, add = FALSE)

distance_to_centroid_overall <- betadisp_all$distances

between <- samples_site%>% mutate(distance = distance_to_centroid_overall) %>%
dplyr::select(distance)
between$distance_type <- "between"


```


#include also body site distance within indiviudals:
```{r}
samples_dorsal <- samples_site %>% filter(body_site=="dorsal")

perch_counts_clr <- read_tsv('perch_counts_clr.tsv', show_col_types = FALSE)
````
````

```
perch_counts_clr <- perch_counts_clr %>% inner_join(samples_dorsal, by ="sample") %>%
dplyr::select(sample, seqid, clr)

perch_euclidean_dorsal <- perch_counts_clr %>%
  dplyr::select(sample, seqid, clr) %>%
  # We need a wide table with clr values, asvs as columns and samples as rows
  spread(seqid, clr) %>%
  tibble::column_to_rownames('sample') %>%
  # The dist functions calculates, by default, euclidian distances between rows in the table
  dist()%>%
  as.matrix()
#For plot: distance based on all samples instead of within individuals
betadisp_dorsal <- betadisper(as.dist(perch_euclidean_dorsal), samples_dorsal$fish_nr,
type = "centroid", bias.adjust = FALSE, sqrt.dist = FALSE, add = FALSE)

distance_to_centroid_dorsal <- betadisp_dorsal$distances

samples_dorsal <- samples_dorsal %>% mutate(distance = distance_to_centroid_dorsal)

samples_dorsal <- samples_dorsal %>% mutate(distance_to_centroid_dorsal =
distance_to_centroid_dorsal) %>% dplyr::select(distance)

samples_dorsal$distance_type <- "dorsal"

#Ventral
samples_ventral <- samples_site %>% filter(body_site=="ventral")

perch_counts_clr <- read_tsv('perch_counts_clr.tsv')

perch_counts_clr <- perch_counts_clr %>% inner_join(samples_ventral, by ="sample") %>%
dplyr::select(sample, seqid, clr)

perch_euclidean_ventral <- perch_counts_clr %>%
  dplyr::select(sample, seqid, clr) %>%
  # We need a wide table with clr values, asvs as columns and samples as rows
  spread(seqid, clr) %>%
  tibble::column_to_rownames('sample') %>%
  # The dist functions calculates, by default, euclidean distances between rows in the table
  dist()%>%
  as.matrix()

#For plot: distance based on all samples instead of within individuals
betadisp_ventral <- betadisper(as.dist(perch_euclidean_ventral), samples_ventral$fish_nr,
type = "centroid", bias.adjust = FALSE, sqrt.dist = FALSE, add = FALSE)

distance_to_centroid_ventral <- betadisp_ventral$distances
```

````
samples_ventral <- samples_ventral %>% mutate(distance = distance_to_centroid_ventral)
%>% dplyr::select(distance)

samples_ventral$distance_type <- "ventral"
```

#Combine distances for Figure 5:
```{r}
data_d <- rbind(samples_dorsal, samples_ventral, within, between )
data_d$distance_type <- factor(data_d$distance_type, levels = c("dorsal", "ventral",
"within", "between"))
library(harrypotter)

distance_within_between_all_levels <- data_d %>% ggplot(aes(x=distance_type,
y=distance)) +
  geom_boxplot(aes(color=factor(distance_type)), outlier.shape = NA) +
  scale_color_hp_d(option = "LunaLovegood")+
  geom_jitter(aes(color=distance_type), size=0.5)+
  #scale_fill_manual(values=c("grey", "white"))+
  theme_classic() +
  theme(legend.position = "bottom")+
 # guide_legend(nrow=2,byrow=TRUE)+
  theme(axis.text.x = element_blank())+
  theme(legend.title = element_blank())+
  theme(axis.title = element_text(size=14)) +
  theme(legend.text = element_text(size = 12))+
  xlab("") +
  ylab("Distance to centroid")

distance_within_between_all_levels #
#ggsave("Figure5.jpg", units = c("mm"), dpi = 300, height = 80, width = 110)
```
#Figure 6

```{r}
#for each individual:
betadisp_ind <- betadisper(as.dist(perch_euclidean_ind), samples_site$fish_ID, type =
"centroid", bias.adjust = FALSE, sqrt.dist = FALSE, add = FALSE)
"boxplot"(betadisp_ind, ylab = "Distance to centroid", xlab = "Fish ID", color =
samples_site$fish_ID)

anova_dispersion_individ <- anova(betadisp_ind)

distance_to_centroid_individ <- betadisp_ind$distances
````

```
samples_site <- samples_site%>% mutate(distance_to_centroid_individ =
distance_to_centroid_individ)

#write_tsv(samples_site, "distance_within_ind.tsv")

distanc_plot_ind_eu <- samples_site %>% ggplot(aes(x=fish_nr,
y=distance_to_centroid_individ)) +
  geom_point(colour="black", shape=21, size = 2.5,
     aes(fill = factor(body_site))) +
     scale_fill_manual(values=c("black", "white")) +
  #scale_color_manual(values=c("black", "grey")) +
  #scale_fill_manual(values=c("#8B008B", "coral"))+
  theme_classic() +
  theme(legend.position = "bottom")+
  theme(legend.title = element_blank())+
  theme(legend.text =  element_text(size = 14))+
  theme(axis.title = element_text(size=14))+
  xlab("Individual") +
  ylab("Distance to centroid - Euclidean") +
  facet_grid(~pop, scales = "free_x", space = "free" )

distanc_plot_ind_eu #Figure 6



```
